# Proteomics analysis identifies PEA-15 as an endosomal phosphoprotein that regulates α5β1 integrin endocytosis

**DOI:** 10.1038/s41598-021-99348-z

**Published:** 2021-10-06

**Authors:** Maisel J. Caliva, Won Seok Yang, Shirley Young-Robbins, Ming Zhou, Hana Yoon, Michelle L. Matter, Mark L. Grimes, Thomas Conrads, Joe William Ramos

**Affiliations:** 1 Cancer Biology Program, University of Hawaii Cancer Center, University of Hawaii at Mānoa, 701 Ilalo Street, Honolulu, HI 96813 USA; 2 Women’s Health Integrated Research Center at Inova, Inova Women’s Service Line, Inova Health System, 3289 Woodburn Rd, Suite 375, Falls Church, VA 22003 USA; 3 Division of Biological Sciences, Center for Structural and Functional Neuroscience, University of Montana, 32 Campus Drive, Missoula, MT 59812 USA

**Keywords:** Cell migration, Integrin signalling, Cell biology, Cell adhesion, Integrins

## Abstract

Endosomal trafficking of cell surface receptors is essential to their function. Integrins are transmembrane receptors that integrate adhesion to the extracellular matrix with engagement of the cytoskeleton. Ligated integrins mediate diverse signals that regulate matrix assembly, cell survival, cell morphology, and cell motility. Endosomal trafficking of integrins modulates these signals and contributes to cell motility and is required for cancer cell invasion. The phosphoprotein PEA-15 modulates integrin activation and ERK MAP Kinase signaling. To elucidate novel PEA-15 functions we utilized an unbiased proteomics approach. We identified several binding partners for PEA-15 in the endosome including clathrin and AP-2 as well as integrin β1 and other focal adhesion complex proteins. We confirmed these interactions using proximity ligation analysis, immunofluorescence imaging, pull-down and co-immunoprecipitation. We further found that PEA-15 is enriched in endosomes and was required for efficient endosomal internalization of α5β1 integrin and cellular migration. Importantly, PEA-15 promotion of migration was dependent on PEA-15 phosphorylation at serines 104 and 116. These data support a novel endosomal role for PEA-15 in control of endosomal trafficking of integrins through an association with the β1 integrin and clathrin complexes, and thereby regulation of cell motility.

## Introduction

Integrins are a family of extracellular matrix (ECM) receptors that are important for a range of physiological and pathological processes including the immune response, wound healing, embryogenesis, and cancer^1,2^. Each integrin is comprised of an alpha and beta heterodimer, and different alpha and beta integrin combinations bind specific ECM proteins^3^. Integrin affinity for these ECM ligands is dependent on the conformational activation state of the integrin heterodimer, which is controlled through inside-out signal transduction by proteins such as Rap1 and RSK2 that signal through talin and Filamin A respectively^4–6^. Integrin binding to ECM initiates outside-in activation of signaling networks involving a large number of proteins that converge at sites of integrin clustering called focal adhesions, where integrins form a dynamic link between the actin cytoskeleton and the extracellular microenvironment^7,8^. Integrins are also subject to constitutive endocytosis and endosomal recycling back to the plasma membrane. Cells can regulate integrin trafficking in response to both intrinsic signaling and extracellular cues^9^. Cells can thereby modulate the composition of different integrin heterodimers at the cell surface, allowing for adaptive adhesive behavior toward the microenvironment. Moreover, aberrant integrin trafficking has been observed in a number of cancer types^10,11^.

Integrin internalization utilizes much of the same trafficking machinery used by receptor tyrosine kinases^12^. In general, endocytosis depends on recruitment of coat proteins like clathrin that induce membrane curvature in concert with endocytic accessory proteins. Adapter proteins link endocytic cargo to these sites, which are then pinched off by dynamin mediated constriction. The majority of integrins avoid lysosomal degradation and are diverted by the small GTPase Rab5 into early endosomes^13^. Exchange of Rab5 for Rab11 is required for recycling of integrins back to the plasma membrane. Although integrin trafficking is constitutive, the rate of recycling can be increased by cell stimulation and is therefore regulated by canonical signaling pathways^14,15^. For example, phosphorylation of the ArfGAP ACAP1 by AKT is required for α5β1 recycling in serum-stimulated cells^16^. The enhanced cell migration and invasiveness of many cancer types can be correlated with overexpression or aberrant regulation of many of these molecules^17,18^.

PEA-15 (Phosphoprotein Enriched in Astrocytes of 15 kDa) is a member of Death Effector Domain (DED) family and regulates cell adhesion by controlling the activity of integrins^19^. PEA-15 has been shown to regulate cell migration in breast cancer, ovarian carcinoma and neuroblastoma^20–22^. In glioma cells, PEA-15 promotes cell survival by blocking low-glucose induced apoptosis^23^. PEA-15 interacts with other DED-containing proteins and with ERK 1/2, and this is regulated by phosphorylation of carboxy-terminal serine residues 104 and 116. PEA-15 therefore engages both proliferative and apoptotic signaling depending on its phosphorylation status^24,25^. In the case of ERK 1/2, PEA-15 functions as a downstream scaffold that targets ERK 1/2 to phosphorylate and activate Ribosomal S6 Kinase 2 (RSK-2)^26^. RSK phosphorylates its substrate Filamin thereby recruiting it to focal adhesions which then leads to suppression of integrin activation and subsequent inhibition of fibronectin (FN) matrix assembly and increased migration^6^.

We used an unbiased proteomic screening approach and found that PEA-15 interacts with integrin complexes and multiple regulators of endocytosis, particularly clathrin and AP-2. We further found that PEA-15 is a novel endosomal protein localized in the Rab5 compartment and that it regulates α5β1 integrin endocytosis. We show that PEA-15 regulates integrin subcellular localization at the Rab5 endosome and interacts with β1 integrin cytoplasmic domains that mediate integrin endocytosis. These data indicate a previously unknown function for PEA-15 as a key endosomal adapter for α5β1 integrin.

## Results

### PEA-15 associates with proteins that regulate endosomal trafficking

**Figure 1:**
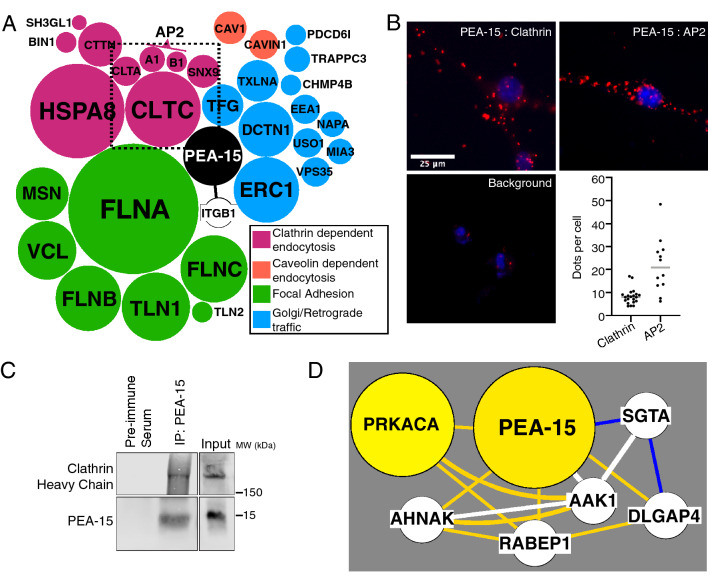
Proteomics analysis reveals PEA-15 interacts with clathrin and AP-2 and integrin focal adhesion proteins. (**A**) Graphical representation of endosomal interacting partners identified in Table 1. PEA-15 (centered black circle) is the immunoprecipitated protein. The size of other circles is proportional to the number of peptides detected by mass spectroscopy from co-immunoprecipitated proteins. These circles are also grouped by function: adhesion (green), clathrin-mediated endocytosis (purple), caveolin-mediated endocytosis (orange), and exocytosis and golgi trafficking (blue). PEA-15 is a known regulator of integrin activity, and this is represented by a line between them. (**B**) The interaction of PEA-15 with clathrin and AP-2 was detected by a Proximity Ligation Assay (PLA). Red dots indicate interaction. Background control shows staining with PEA-15 antibodies alone. Scale bar = 25 µm, DAPI (blue) nuclear counter stain. The average number of red dots per cell was quantified using ImageJ and is shown as scatter plots with a line marking the median. Each point on the scatter plot is an average measure from a field of cells. PLA clathrin: n = 21 fields (601 cells). PLA AP-2: n = 12 fields (194 cells). PLA with antibodies only against PEA-15 was performed for the negative control. (**C**) U87MG lysate was used for immunoprecipitation of PEA-15 with anti-PEA-15 antibodies. Co-precipitated clathrin heavy chain was detected by Western blot. (**D**) Proteins that connect to PEA-15 in a cluster-filtered network of protein–protein interactions and a co-cluster correlation network as shown previously^28^. The size and color of nodes is scaled to graph total phosphopeptides detected for each protein. White lines show proteins that co-cluster and are known to interact. Yellow lines connect positively correlating proteins; blue lines connect negatively correlating proteins.

PEA-15 modulates cell signaling and motility in a variety of cell types^20–22,27^. PEA-15 specifically modulates oncogenic H-Ras mediated inactivation of integrins by a poorly defined mechanism^19^. To identify novel interacting partners and potential new functions of PEA-15 we employed an unbiased proteomics approach. Endogenous PEA-15 was immunoprecipitated from U87MG cell lysates and analyzed by mass spectrometry to identify co-precipitated proteins. As a secondary approach to confirm and potentially extend the proteomic analysis we also transfected HA-tagged PEA15 into U87MG cells and immunoprecipitated this with the anti-HA antibody from lysate. IgG alone was used as control. Thus, we analyzed proteins in complex with PEA15 in these cells using two independent immunoprecipitation methods. Initial analysis was focused on proteins that were detected in both screens (Supp. Table 1, Supp. Table 2). Proteomic analysis identified β1 integrin and focal adhesion proteins complexed with PEA-15 suggesting that PEA-15 interaction with integrin complexes may be important for the effects on migration (Table 1). Surprisingly, we also identified an additional subset of co-precipitated proteins with known functions in endocytosis and endosomal trafficking (Table 1, Fig. 1A). In particular, we detected clathrin (both light and heavy chains) and AP2 (Alpha and Beta subunits). Both proteins are required for recruitment of surface cargoes in coordination with clathrin lattice formation on the cytoplasmic face at endocytic pits. To determine if these proteins interact in intact cells, we did proximity ligation analysis (PLA) visualizing endogenous protein interactions. In support of the proteomics data, we detected positive signal by PLA between PEA-15 and both clathrin and AP2 (Fig. 1B). We further confirmed co-immunoprecipitation of clathrin with PEA-15 by Western blot (Fig. 1C).

**Table 1:** Unbiased proteomic screen of PEA-15 co-immunoprecipitation.

Protein	Gene	Accession	Peptides	Peptide Coverage	Regulatory Function
Clathrin heavy chain 1	*CLTC*	Q00610	27	16	Coat protein
Clathrin light chain A	*CLTA*	P09496	4	10	Coat protein
AP-2 complex subunit alpha-1	*AP2A1*	O95782	3	7	Adapter
AP-2 complex subunit beta	*AP2B1*	P63010	2	0	Adapter
Heat Shock 70 kDa Protein 8	*HSPA8*	P11142	42	45	ATPase, uncoating
Sorting nexin-9	*SNX9*	Q9Y5X1	5	9	Dynamin recruitment
Cortactin	*CTTN*	Q14247	9	21	Actin at CCPs
Amphiphysin II (Myc box-dependent-interacting protein 1)	*BIN1*	O00499	2	4	Membrane Curvature
Endophilin-A2	*SH3GL1*	Q99961	1	3	Membrane Curvature
Caveolin-1	*CAV1*	Q03135	5	30	Coat protein
Caveolae-associated protein 1	*CAVIN1*	Q6NZI2	4	12	Caveolin coat scaffold
ELKS/Rab6-interacting/CAST family member 1	*ERC1*	Q8IUD2	20	20	Trafficking in neurotransmitter release
Dynactin subunit 1	*DCTN1*	Q14203	14	10	Motor protein tether, Golgi Transport
Protein TFG	*TFG*	Q92734	8	20	ER—Golgi traffic
Alpha-taxilin	*TXLNA*	P40222	8	16	Calcium-stimulated Exocytosis
Early endosome antigen 1	*EEA1*	Q15075	4	3	Endosomal trafficking
Vacuolar protein sorting-associated protein 35	*VPS35*	Q96QK1	4	7	Endosomal trafficking, retrograde to golgi
General vesicular transport factor p115	*USO1*	O60763	4	5	Golgi traffic
Alpha-soluble NSF attachment protein	*NAPA*	P54920	3	14	ER—golgi traffic, membrane fusion
Trafficking protein particle complex subunit 3	*TRAPPC3*	O43617	3	13	Golgi traffic
Transport and Golgi organization protein 1 homolog	*MIA3*	Q5JRA6	3	1	Golgi traffic
Charged multivesicular body protein 4b	*CHMP4B*	Q9H444	2	8	Trafficking to multivesicular bodies
Programmed cell death 6 -interacting protein	*PDCD6IP*	Q8WUM4	2	2	Trafficking to multivesicular bodies
Moesin	*MSN*	P26038	13	21	Focal adhesion
Filamin-A	*FLNA*	P21333	80	30	Focal adhesion
Filamin-B	*FLNB*	O75369	20	10	Focal adhesion
Filamin-C	*FLNC*	Q14315	21	12	Focal adhesion
Talin-1	*TLN1*	Q9Y490	22	12	Focal adhesion
Talin-2	*TLN2*	Q9Y4G6	2	0	Focal adhesion
Vinculin	*VCL*	P18206	16	16	Focal adhesion
Integrin beta-1	*ITGB1*	P05556	4	6	Focal adhesions

PEA-15 was immunoprecipitated from lysates of U87MG. Co-immunoprecipitants were detected by mass spectroscopy and proteomic analysis. Shown is a subset of identified proteins that includes known regulators of endosomal traffic. Proteins are categorized by function and then by number of detected peptides. All proteins listed here were also detected in a parallel experiment with cells overexpressing HA-tagged-PEA-15 and immunoprecipitation with anti-HA antibodies.

A third independent proteomics screen was done using neuroblastoma-derived cell lines to detect AKT-phosphorylated proteins enriched in endosomes. In this approach endosomes were isolated from permeabilized neuroblastoma-derived cells by velocity sedimentation and flotation equilibrium centrifugation^29^ and then extracted for protein. AKT substrate immunoprecipitations were prepared for MS analysis. PEA-15 was identified in this screen to be phosphorylated by AKT at serine 116 in endosomes from three neuroblastoma cell lines (LAN-6, SMS-KCN, SK-N-BE(2) (Supplemental Fig. 1). PEA-15 was also one of the most highly phosphorylated proteins detected in neuroblastoma endosomes (fourth topmost phosphorylated protein in endosomes) (Supplemental Fig. 1). Clustering analysis^28^ demonstrated that phosphorylation of PEA-15 was most highly correlated with that of PRKACA (protein kinase A catalytic alpha subunit) and also commonly phosphorylated with several other proteins, suggesting a functional network (Fig. 1D). This group includes the AP2 Associated Kinase 1 (AAK1) which regulates clathrin-dependent endocytosis^30^. Rabaptin (RABEP1) is also found in the PEA-15 group and is a Rab5 effector that regulates early endosome fusion^31^.

**Figure 2:**
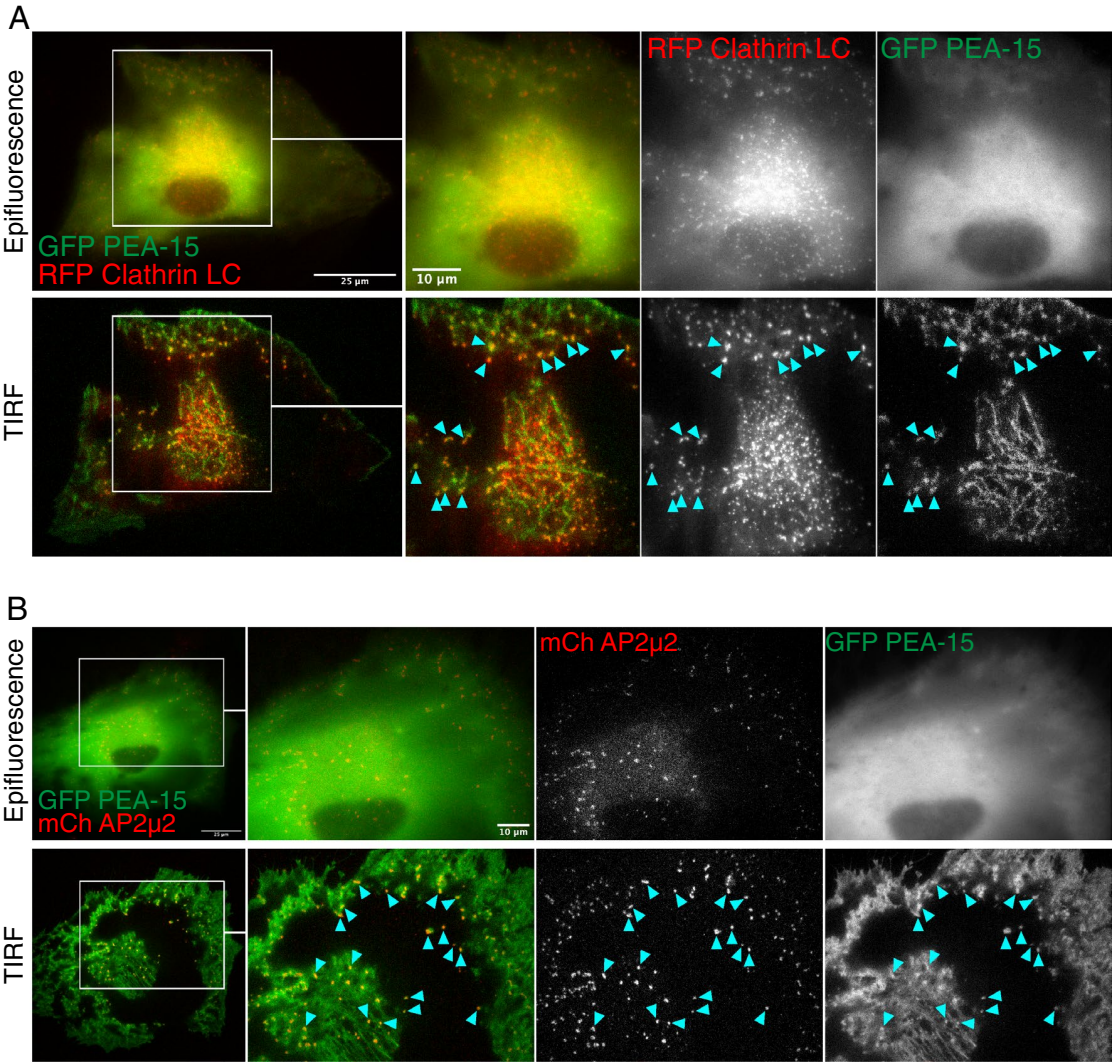
PEA-15 is localized to Clathrin and AP-2 at the plasma membrane. (**A**) GFP-tagged PEA-15 and RFP-tagged Clathrin light chain were co-expressed in U87MG cells and observed under both epifluorescent and TIRF (Total Internal Reflection Fluorescence) imaging. (**B**) Images were acquired as in part A, but instead with GFP-PEA15 coexpressed with mCherry-tagged AP2µ2. Blue arrows indicate sites of colocalization at the cell surface resolved in TIRF. Scale bar in zoomed-out images = 25 µm. Scale bar in zoomed-in images = 10 µm**.**

PEA-15 has been characterized previously as a cytoplasmic protein. The proteomic data indicated that PEA-15 may be associated with Clathrin-enriched sites in the cell. To further confirm the proteomic data, we tested whether PEA-15 is enriched at specific sites of the plasma membrane. GFP-tagged PEA-15 was co-expressed with RFP-tagged Clathrin light chain (LC), which is used to fluorescently mark clathrin-coated pits during live imaging^32^. We observed significant overlap of PEA-15 and Clathrin LC in the perinuclear region (Fig. 2A). To limit detection of signal to the plasma membrane, we observed the cell using TIRF (Total Internal Reflection Fluorescence). Under TIRF imaging, PEA-15 appeared in defined spots near the cell edge that colocalized with Clathrin, while at the interior of the cell, especially under the nucleus, PEA-15 appeared to have a tubular morphology intermittently colocalized with Clathrin (Fig. 2A). We similarly assayed the colocalization of PEA-15 with AP2 using mCherry-tagged AP2µ2, another marker for AP2 used in live imaging^33^. Using TIRF, we observed colocalization of PEA-15 with AP2 in defined spots at the cell membrane (Fig. 2B). No clear colocalization was observed between control GFP and either Clathrin LC or AP2µ2 (Supplementary Fig. 2).

**Figure 3:**
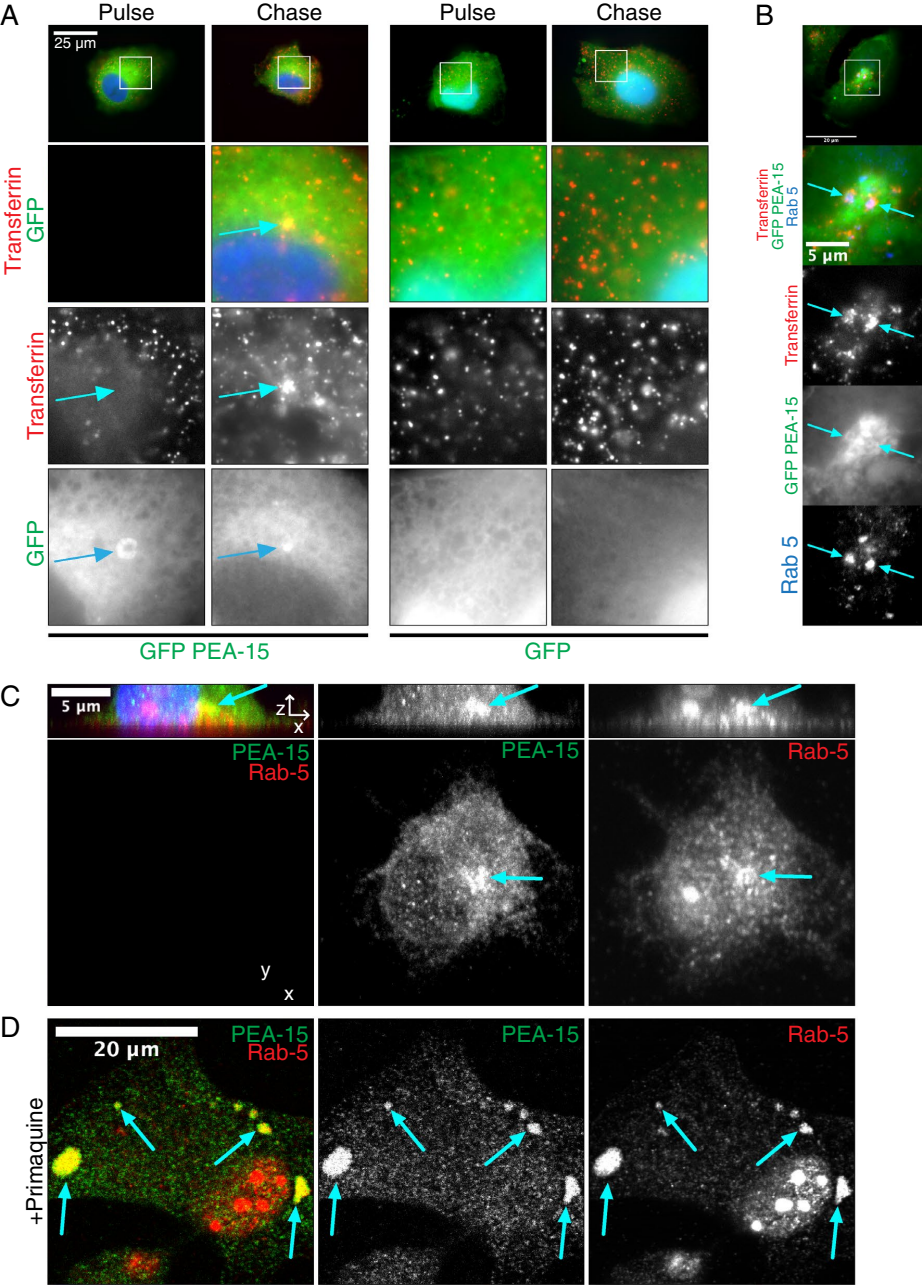
PEA-15 co-traffics with Transferrin. (**A**) HeLa cells expressing GFP-tagged PEA-15 were incubated with Rhodamine-Transferrin for 2 min at 37 °C (Pulse). Non-internalized Transferrin was washed away before incubation at 37 °C in the presence of unlabeled transferrin (Chase). Immunofluorescence imaging shows the co-trafficking of GFP-PEA15 and chased Rhodamine-Transferrin to the perinuclear region. No trafficking was observed with GFP control. Scale bar in zoomed-out images = 25 µm. (**B**) Cells were processed as in Part A, but were also immunostained with antibodies against Rab5. Scale bar in zoomed-out images = 20 µm. Scale bar in zoomed-in images = 5 µm. (**C**) U87MG cells attached to fibronectin-coated glass coverslips for 30 min before co-immunostaining for endogenous PEA-15 and endogenous Rab5. Shown are maximum intensity projections from z-stacks acquired on a confocal microscope (XY) paired with orthogonal view (XZ). Arrows indicate perinuclear colocalization of PEA-15 with Rab5. (**D**) U87MG cells plated in normal growth conditions with the addition of primaquine were co-immunostained for endogenous PEA-15 and endogenous Rab5. Arrows indicate colocalization.

The data suggested that PEA-15 is enriched at Clathrin-coated pits and may have a novel role in endocytic trafficking. We next assessed whether PEA-15 can co-traffic with Transferrin. Transferrin functions as an iron transporter that is endocytosed via the Transferrin Receptor. Fluorescently-tagged Transferrin given to cells in culture is used to label the endosomal compartment^34^. We pulsed GFP-PEA15 expressing HeLa cells briefly with Rhodamine-Transferrin, washed, then chased the Rhodamine-Transferrin with unlabeled Transferrin. Fluorescence imaging showed that pulsed Rhodamine-Transferrin trafficked to the perinuclear region and colocalized there with GFP-PEA15 (Fig. 3A). This was not observed in cells expressing GFP. We next immunostained the same cells with antibody against the early endosome marker Rab5.

Imaging revealed that the sites of colocalization between PEA-15 and Transferrin were positively stained for Rab5, which supports that PEA-15 and Transferrin co-traffic to the early endosome (Fig. 3B).

**Figure 4:**
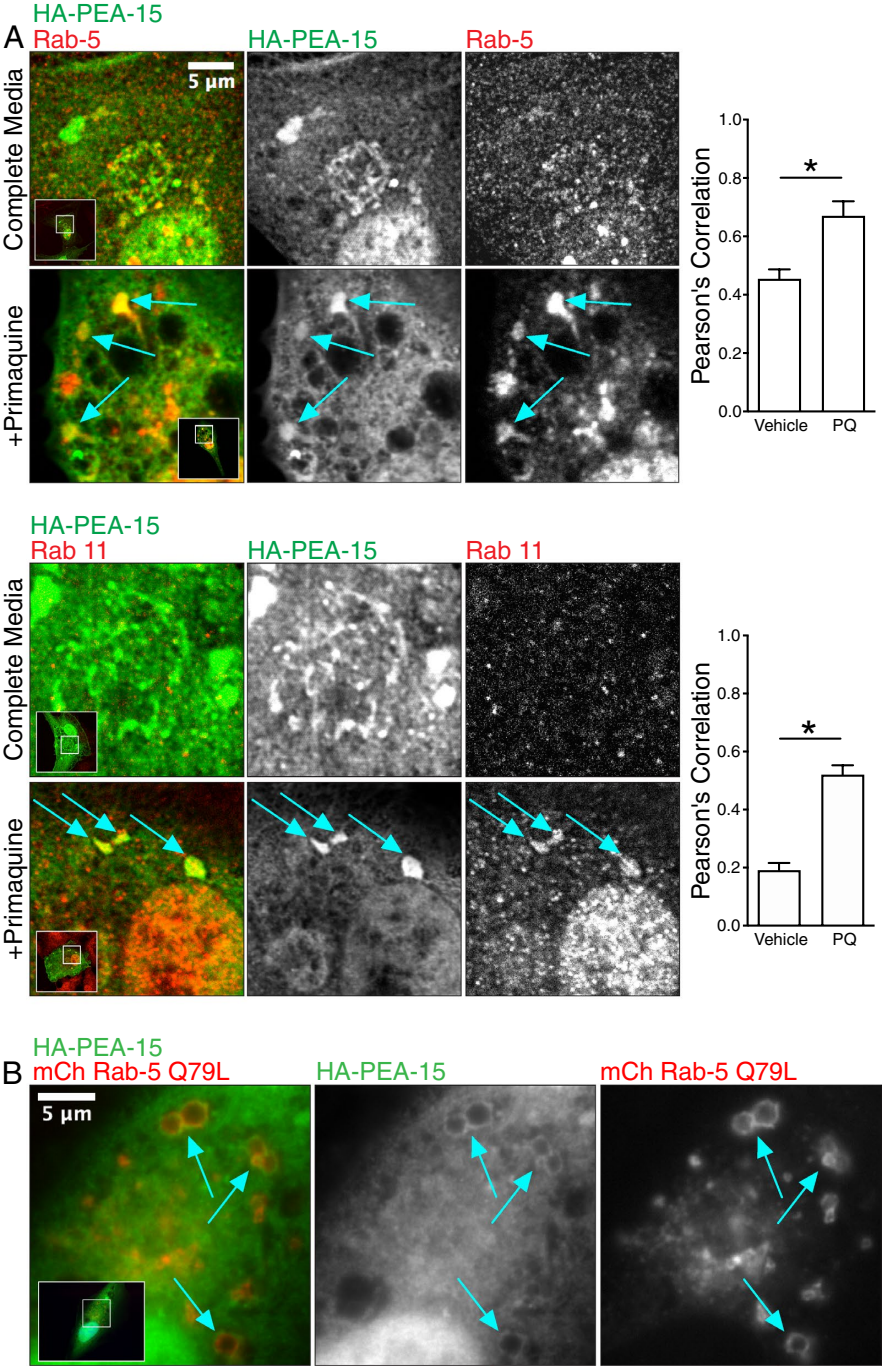
PEA-15 traffics to Rab5 endosomes. (**A**) HA-tagged PEA-15 was expressed in U87MG cells that were treated with primaquine or control. Cells were then fixed with paraformaldehyde and immunostained for PEA-15, Rab5 or the recycling endosome marker Rab11. Co-localization between PEA-15 and endosomal markers was measured by quantitation of the Pearson correlation using ImageJ software (*p < 0.005, n = 11 for each pairing). Enlarged vesicles co-staining for both HA-tagged PEA-15 and endosomal markers are indicated by arrows. Scale bar = 5 µm. (**B**) HA-tagged PEA-15 and mCherry-tagged Rab5 Q79L mutant were overexpressed in U87MG cells and immunostained with anti-HA antibodies for PEA-15. Co-localization of HA-PEA-15 and mCherry Rab5 Q79L occurred on ringed structures (arrows). Scale bar = 5 µm.

We next determined whether PEA-15 co-localizes with known endosomal markers in U87MG cells. Under normal culture conditions PEA-15 did not show clear co-immunostaining with endosomal markers. However, when U87MG cells were briefly adhered to fibronectin for 30 min, we observed endogenous PEA-15 concentrating in a perinuclear Rab5 early endosome compartment (Fig. 3C). The association of endosomal proteins with vesicles is typically weak and transient^35^. Primaquine has been used previously to inhibit endosomal recycling and increase the size and stability of endosomes^36–39^. After treating U87MG cells in normal growth conditions with primaquine we again observed endogenous PEA-15 enriched in Rab5 endosomes (Fig. 3D). HA-tagged PEA-15 expressed in U87MG showed modest co-immunostaining with Rab5 early endosomes and Rab11 recycling endosomes under normal growth conditions (Fig. 4A). Upon primaquine treatment, we observed enlarged Rab5 vesicles that also showed significant co-localization with HA-tagged PEA-15, and this was also demonstrated when co-staining for Rab11 (Fig. 4A).

To compliment this approach we expressed the constitutively active Rab5 Q79L mutant, which prevents Rab5 dependent endosomal fission and increases the rate of endosomal fusion^40^. This promotes formation of enlarged and stabilized endosomes that appear as rings. In U87MG cells expressing both HA-tagged PEA-15 and mCherry-tagged Rab5Q79L, we observed co-localization on these ring-like structures (Fig. 4B). Taken together, these data support subcellular localization of PEA-15 at endosomes.

### PEA-15 interacts with β1 Integrin and is required for cell motility

**Figure 5:**
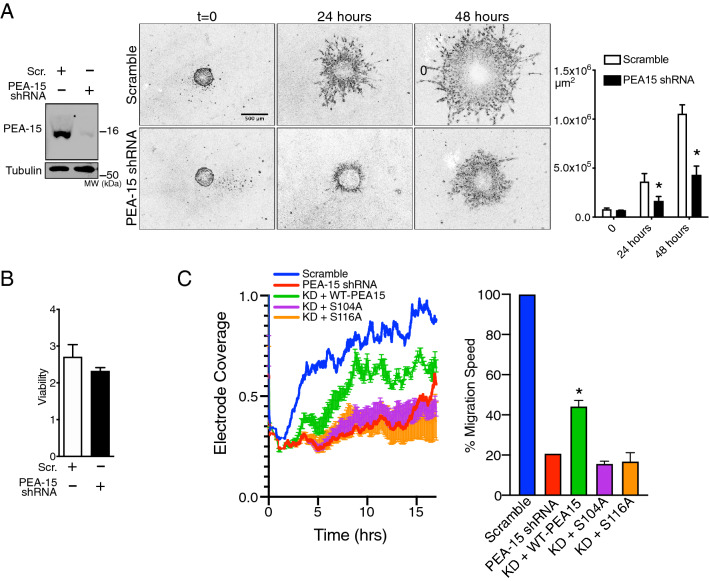
PEA-15 is required for cell migration. (**A**) A pool of shRNAs were used to generate stable knock down of PEA-15 expression in U87MG. PEA-15 KD and scramble control spheroid invasion into a 3D matrix was then followed over time (Scale bar = 500 µm). Invasion by the cell mass was quantified with ImageJ (*p < 0.05, n = 3). (**B**) The viability of scramble and PEA-15 KD cells was measured by XTT assay (signal normalized to background, n = 3, no significant differences). (**C**) ECIS chambers were used to measure wound recovery overtime in KD cells transfected with wild type PEA-15, the S104A mutant, or the S116A mutant. Rescue of migration in KD cells was only detected in cells transfected with wild type PEA-15 (WT vs S104A: *p < 0.005, n = 4; WT vs S116A: *p < 0.005, n = 4).

Location of PEA-15 at the endosome suggests that it may have unknown functions in endosomal trafficking. Along with the endosomal proteins we identified integrins, including integrin β1, in association with PEA-15. Given that PEA-15 regulates integrins and cellular adhesion^19^, we hypothesized that PEA-15 regulates endocytosis and endosomal recycling of integrins in cellular invasion. To test this, we generated a stable shRNA knockdown of PEA-15 in U87MG cells and assayed their invasiveness in 3D culture. Knockdown of PEA-15 caused a significant decrease in invasiveness while having no effect on cell proliferation (Fig. 5A,B). PEA-15 knock down cells also exhibited decreased migration in 2D scratch assays, and this migration was rescued by re-expression of PEA-15 (Supplemental Fig. 3).

We tested the role of phosphorylation of PEA-15 in cell migration by expressing mutants with a serine-to-alanine substitution at position 104 or 116 in the PEA-15 KD cells. The cells were grown to confluence over the electrode of ECIS (Electric Cell-Substrate Impedance Sensing) chambers. ECIS has been used previously to quantify cell migration in U87MG cells^41^. Shock wounding creates a wound over the electrode, allowing for the movement of cells back onto the electrode to be detected as an increase in impedance over time. Scramble control cells were able to move and cover most of the electrode area (~ 75%) within 10 h after wounding. PEA-15 KD cells only reoccupied approximately 50% of the electrode area past 15 h, indicating less cell motility in the KD cells (Fig. 5C). In agreement with our scratch assay, KD cells transfected with wildtype PEA-15 moved over the electrode at a higher rate compared to control KD cells, indicating partial rescue of cell motility. This was not seen in KD cells re-expressing S104A or S116A mutants. Relative cell velocities estimated from ECIS data showed significantly higher speed in the wildtype rescue cells. Taken together, these migration assays all suggested that PEA-15 expression is required for cell migration and that this is dependent on phosphorylation at both serines 104 and 116.

**Figure 6:**
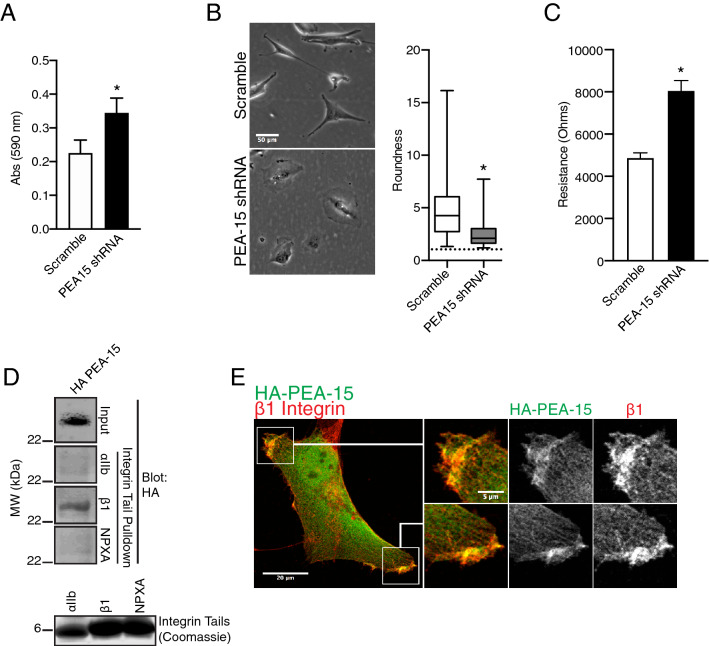
PEA-15 interacts with β1 Integrin. (**A**). U87MG PEA-15 knock down cells were plated on Fibronectin-coated plates. The number of adhered cells was then quantified by crystal violet staining and is shown as absorbance at 590 nm (n = 4, *p* < 0.005). (**B**) Scramble and PEA-15 KD were cells grown under normal growth conditions for 24 h in plastic tissue culture dishes. Bright-field images were acquired and cell area and perimeter were recorded and used to calculate a roundness index (the dotted line indicates a value of 1, corresponding to a perfectly circular morphology). PEA-15 KD cells displayed a significantly rounder and spread morphology compared to control cells (*p < 0.005, n = 150). Scale bar = 50 µm. (**C**) Equal numbers of scramble and PEA-15 KD cells were seeded then grown to confluence in ECIS chambers. The baseline resistance of PEA-15 KD cells at confluence is significantly increased compared to scramble control (*p < 0.005, n = 4). (**D**) Recombinant β1 integrin cytoplasmic tails were incubated with lysate from HeLa cells expressing HA-tagged PEA-15. NPXA = β1 membrane proximal tyrosine mutated to alanine, αIIb = αIIb integrin cytoplasmic tail. HA-PEA-15 was detected in precipitates by Western blot. (**E**) HA-PEA-15 was expressed in PEA-15 KD cells. Cells were fixed and immunostained for HA tag and β1 integrin. Scale bar = 20 µm; zoomed scale bars = 5 µm.

Adhesion on fibronectin was significantly increased in PEA-15 knock down cells, indicating that the effects of PEA-15 on migration may be through fibronectin receptors such as α5β1 integrin (Fig. 6A). PEA-15 KD cells also exhibited a thinly spread morphology compared to scramble controls (Fig. 6B). The baseline impedance of PEA-15 KD cells is significantly increased in confluent cell layers as detected on ECIS electrodes, again indicating a general effect on cell adhesion in the KD cells (Fig. 6C). We further assayed PEA-15 association with integrin complexes using purified integrin tail domains attached to beads in a pulldown approach. We found that HA-tagged PEA-15 was precipitated by recombinant β1 integrin cytoplasmic tail domain (Fig. 6D). This supports close association of PEA-15 with the β1 integrin cytoplasmic domain. Importantly, PEA-15 failed to interact with the β1 cytoplasmic tail when its membrane-proximal NPxY tyrosine is mutated to alanine (NPxA). This residue is important for talin binding to β integrins, integrin activation, and integrin internalization^42^. In alignment with these pulldown data, we also found colocalization of PEA-15 and β1 integrin at the front of cell protrusions in U87MG cells, thereby supporting a role for this complex in motility (Fig. 6E).

### PEA-15 sorts β1 integrin to early endosomes

**Figure 7:**
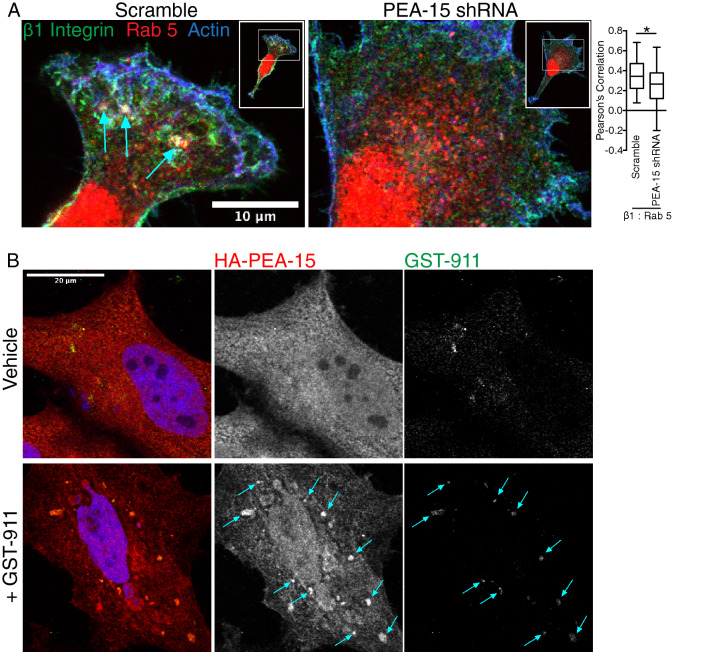
PEA-15 sorts β1 integrins to Rab5 endosomes. (**A**) Scramble and PEA-15 KD cells were allowed to adhere to FN-coated glass coverslips for 30 min. Cells were fixed and stained for β1 integrin, Rab5, and actin. Scale bar = 10 µm. Areas showing overlap between all three channels are indicated by white arrows. The Pearson correlation between β1 integrin and Rab5 endosomes was measured using ImageJ software (n = 37, *p < 0.05). (**B**) HeLa cells expressing HA-tagged PEA-15 were treated with or without GST-3FN9-11 (GST-911) and co-stained with antibodies against HA-tag and GST. Vesicular structures showing co-localized signal are indicated by blue arrows. Scale bar = 20 µm.

Because the data shows association of PEA-15 with both β1 integrin and Rab5 endosomes, we next tested whether PEA-15 expression regulates the association of β1 integrin with Rab5 endosomes. U87MG PEA-15 knockdown cells were plated on fibronectin-coated glass slides followed by co-immunostaining for β1 integrin and Rab5. Scramble control cells showed β1 integrins concentrated at the leading edge and co-localized with both actin and Rab5 at vesicles immediately behind the leading edge (Fig. 7A). In contrast co-localization of β1 integrin with Rab5 was consistently and significantly reduced in PEA-15 knock down cells, where instead β1 integrin was evenly dispersed throughout the cytoplasm and did not exhibit enrichment at the cell front. To additionally test whether PEA-15 co-traffics with ligated integrins on endosomal vesicles, we treated cells with a recombinant soluble fibronectin fragment that includes the RGD ligand sequence and which binds predominantly to α5β1 integrin (GST-3FN9-11)^43^. While PEA-15 exhibited an even distribution throughout the cytoplasm in untreated cells, GST-3FN9-11 treated cells caused HA-tagged PEA-15 to form large vesicles that were also enriched with the GST-3FN9-11 signal (Fig. 7B). This suggests that FN binding to α5β1 integrins induces localized recruitment of PEA-15 to the integrin endosomal compartment. Taken together, the preceding data suggest that PEA-15 may function in the endosomal internalization or recycling of integrins.

### PEA-15 regulates α5β1 integrin internalization

**Figure 8:**
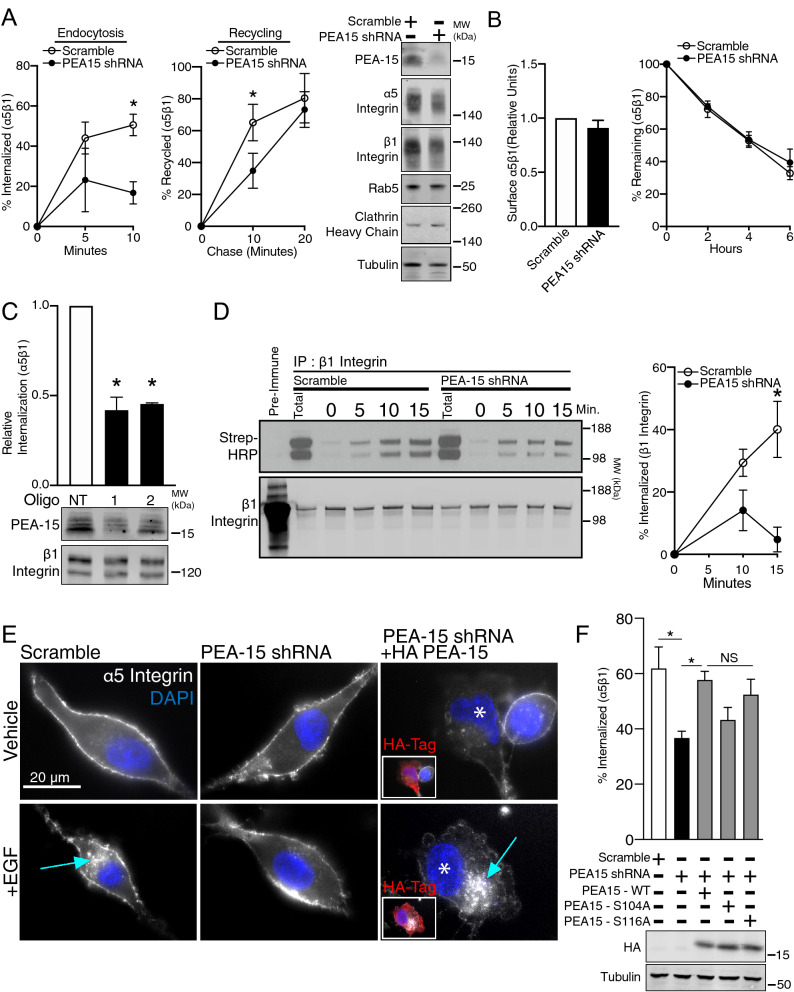
PEA-15 regulates integrin trafficking. (**A**) Strip-resistant (internalized) biotinylated integrins were quantified by capture ELISA with antibodies against α5 integrin and expressed as a percentage of surface integrin absorbance signal. Shown is internalization after 5 min (*p* = 0.2299, n = 2) and 15 min (*p < 0.05, n = 5) in the PEA-15 knock down cells compared to scramble control. Chased (recycled) biotinylated integrins were also quantified by capture ELISA. Shown is the percent α5 integrin recycled relative to pulse controls at 10 min (*p < 0.005, n = 4) and 20 min (*p* = 0.5452, n = 3). Western blots show expression of α5 integrin, β1 integrin, Rab5 and Clathrin Heavy Chain, in the stable PEA-15 KD cells. (**B**) Surface biotinylated integrin α5β1 was quantified by capture ELISA (normalized absorbance to scramble control, *p* = 0.2439, n = 7). Degradation of surface α5 integrin was quantified over 6 h. Shown is the percent signal compared to time 0 (n = 3). (**C**) Integrin endocytosis was quantified in U87MG with two independent siRNAs (1 and 2). Shown is internalization normalized to non-target (NT) control (*p < 0.05, n = 3 for siRNA 1, n = 2 for siRNA 2). (**D**) Integrin endocytosis was measured using immunoprecipitation with antibodies against β1 integrin and detection with Western blotting with Streptavidin-HRP. Shown is a representative Western blot and internalization time course with points at 10 min (*p* = 0.2425, n = 3) and 15 min (*p < 0.05, n = 3). (**E**) Surface α5β1 integrin was labeled with mouse anti-α5 antibodies in PEA-15 KD cells and scramble controls on ice. Cells were then returned to 37 °C in the presence of EGF to induce integrin trafficking, followed by fixation and immunostaining. HA-tag PEA-15 expressed in a subset of PEA-15 KD cells were co-stained with anti-HA-tag antibodies. Asterisks (*) mark transfected cells. Inset images show red-fluorescent HA-tag signal. Scale bar = 20 µm. (**F**) Integrin endocytosis was quantified in PEA-15 KD cells expressing HA-tagged PEA-15 (*p < 0.05, Scramble n = 3, PEA-15 shRNA n = 4, HA-PEA15 rescue n = 3, HA-S104A n = 3, HA-S116A n = 3).

To specifically measure α5β1 integrin internalization and recycling, we used an established cell surface biotinylation and capture ELISA approach using antibodies against α5 integrin^14^. PEA-15 knock down cells exhibited a significant decrease in α5β1 integrin internalization (Fig. 8A). We also measured a significant decrease in α5β1 integrin recycling, although recycling in these cells matches control at longer time points. This indicates a lag in the recycling pathway rather than complete inhibition. We did not detect an effect on either the surface expression or degradation rates of α5β1 in the PEA-15 knock down cells (Fig. 8B). To control for potential off-target or cell line specific effects of the shRNA we further determined how knock down of PEA-15 expression with two independent siRNAs affected internalization and found this likewise resulted in decreased internalization of α5β1 (Fig. 8C). In a third approach we followed internalization of biotinylated β1 integrin by Western blot. In agreement with the α5 integrin capture ELISA approach, we measured a significant decrease in β1 integrin internalization in the PEA-15 knock down cells (Fig. 8D). To visualize the effect of PEA-15 on α5β1 internalization, we labeled the surface of unfixed U87MG cells with anti-α5 integrin antibodies and followed their localization with and without EGF stimulation (Fig. 8E). In control cells, we observed a cohort of α5β1 integrin internalized and trafficked to the perinuclear region in response to EGF (Fig. 8E, Scramble). In contrast, we saw no response in PEA-15 knock down cells, consistent with the biotinylation-ELISA approach. Re-expression of HA-tagged PEA-15 in the knock down cells rescued α5β1 internalization, further demonstrating the requirement for PEA-15. Importantly, we also detected increased α5β1 internalization by capture ELISA when PEA-15 is re-expressed in the knockdown cells (Fig. 8F). In addition, α5β1 internalization was also rescued with expression of either the S104A (lower value but not statistically significant) or S116A mutant, suggesting that phosphorylation at those sites is not required for PEA-15 to drive endocytosis of the integrin. Thus, using multiple detection methods, we find that targeted reduction of PEA-15 expression reduces integrin endocytosis that is rescued by re-expression of PEA-15 indicating it is PEA-15 specific.

## Discussion

Our data demonstrate a novel functional role of PEA-15 in the endosomal trafficking of integrins. This was unexpected given that the previously known functions of PEA-15 in cell adhesion, proliferation, and cell death did not point to an endosomal function. We show PEA-15 binds to proteins associated with the β1 integrin focal adhesion complex and also the clathrin associated endosomal protein complex. We further report that PEA-15 localizes with integrins and endosomal proteins within the cell and regulates endosomal internalization of the integrin complex. The functional consequence of this is reduction of integrin mediated migration and invasion.

Unbiased proteomics screening for PEA-15 binding proteins showed that PEA-15 binds to several proteins at clathrin-coated pits (CCPs) (Fig. 1A). The clathrin heavy chain was among the strongest signals. Also detected in these screens were alpha-adaptin (AP2A1) and beta2-adaptin (AP2B1), which together form binding sites for endocytic adapter and clathrin recruitment to the AP-2 complex^44,45^. Many related endosomal proteins were additionally detected including sorting nexin-9 (SNX9), which is required for recruitment of dynamin at the late stages of CCP maturation^46^, amphiphysin 2 (BIN1) and endophilin A2 (SH3GL1), both regulators of membrane curvature at CCPs^47–49^, the chaperone protein Hsc70 (HSPA8), which has an ATPase function in clathrin coat disassembly after endocytosis^50^, and cortactin (CTTN), which regulates actin assembly at CCPs^51^. These data in combination with proximity ligation analysis and immunofluorescence confirmed PEA-15 localization at CCPs and in Rab5 rich compartments and thereby strongly localize PEA-15 to sites of endocytosis and trafficking. Initiation and progression of CCP formation is a regulated and hierarchical process where CCP proteins are recruited in a step-wise manner^52^. Precisely when PEA-15 is recruited after CCP initiation will need to be determined to understand whether PEA-15 functions as part of an endocytic checkpoint during CCP maturation.

Several focal adhesion proteins were detected in the proteomics screen, including β1 integrin, talin, and filamin. Although PEA-15 is a known regulator of adhesion and migration, this is the first report of PEA-15 interacting in complex with focal adhesion proteins. Importantly this is also the first report of PEA-15 interacting with β1 integrin via the NPXY motif, a motif that is bound by integrin endocytic adapters Dab-2 and Numb^53–56^. Moreover, we find that PEA-15 depletion alters integrin association with endosomes and decreases cell migration and further reduces integrin internalization. PEA-15 also co-traffics with ligand bound α5β1 integrin (Fig. 7B). We therefore propose that PEA-15 is a novel focal adhesion protein that acts as an endocytic adapter for α5β1 integrin. Importantly, phosphorylation of PEA-15 at serines 104 and 116 was not required for PEA-15 rescue of internalization despite phosphorylation being required for rescue of migration (Figs. 5C, 8F). The residues on PEA-15 that regulate endocytosis are therefore distinct from those that regulate migration. Future studies are needed to test other PEA-15 residues, particularly at the flexible C-terminal tail, for endocytic activity. Additionally, given that PEA-15 associates with the NPXY sequence of β1 integrin, it will be important to test whether PEA-15 affects the trafficking of other transmembrane receptors with NPXY motifs. The role of PEA-15 in endocytosis is likely both cargo specific as well as cell type specific.

Proteomics analysis also detected Rabaptin (RABEP1), a direct effector of Rab5 that is required for early endosome fusion^31^. Immunofluorescence imaging supports this demonstrating PEA-15 is concentrated at the Rab5 endosome. PEA-15 co-traffics with internalized α5β1, and sorting of β1 integrin to Rab5 vesicles was decreased in cells where PEA-15 expression was reduced. This suggests that PEA-15 remains associated with newly endocytosed integrins as they transition to the Rab5 compartment. Internalized integrins exhibit distinct signaling patterns compared to integrins at focal adhesions. Recent reports show that Focal Adhesion Kinase (FAK) remains in a complex with integrins after endocytosis and begins signaling in feedback loops with early endosomal proteins, resulting in regulation of anoikis and FAK-dependent migration^57,58^. Given the known signaling scaffold function of PEA-15^59^, it will be important to test whether PEA-15 functions as a scaffold at the endosome and if it has a role in endosomal integrin signaling.

Overall, these data support PEA-15 localization to endosomes, association with both endosomal and focal adhesion proteins, and regulation of endosomal internalization and recycling of integrins in support of cell motility. Furthermore, PEA-15 is required for α5β1 integrin endocytosis and routing to Rab5 early endosomes. This is a novel function for PEA-15 and provides insight into how integrins traffic to the early endosome.

## Methods

### Cell lines and reagents

U87MG human glioblastoma-derived cells were purchased from the NCI-Tumor Repository, Frederick. HeLa cells were purchased from the American Type Culture Collection (ATCC, Manassas, VA). U87MG, and HeLa cells were grown in DMEM (Mediatech, Manassas, VA) supplemented with 10% fetal bovine serum (Gibco, Carlsbad, CA), MEM non-essential amino acids, and penicillin/streptomycin antibiotic. All cell lines were grown in a humidified incubator with a 5% CO_2_ atmosphere at 37 °C. Cell lines were validated by STR profiling by ATCC and tested for mycoplasma (LT07-188; Lonza). All transient transfections of plasmids were conducted with GeneJuice Transfection Reagent (EMD Millipore, Billerica, MA).

PEA-15 antibodies (sc-166678; Santa Cruz Biotechnology, Dallas, TX) were used for immunofluorescence; Western blot detection of endogenous PEA-15 was done with rabbit antibodies produced by Pacific Immunology (Ramona, CA), Phospho-S104 and S116 PEA-15 antibodies were produced by Biosource and described previously^24^, Rab5 (R4654, Sigma-Aldrich, St. Louis, MO), Rab11 (ab65200, Abcam, Cambridge, MA), HA tag (sc-7392 and sc-805, Santa Cruz Biotechnology), α-Tubulin (sc-32293 and sc-12462-R, Santa Cruz Biotechnology), β1 integrin (sc-13590 and sc-8978, Santa Cruz Biotechnology), and GST tag (sc-374171, Santa Cruz Biotechnology). Mouse antibodies against α5 integrin (#555651, BD Biosciences, San Jose, CA) were used for capture ELISA of α5β1 integrin in endocytosis assays, Western blots, and surface labeling for α5β1 integrin visualization by immunofluorescence. Rabbit antibodies against clathrin heavy chain (#4796, Cell Signaling, Danvers, MA) were used in proximity ligase assays and Western blots. Mouse antibodies against Alpha-adaptin were used in proximity ligase assays (#MA1-064, Thermo Fisher, Waltham, MA).

### Mass spectrometry analysis of immunoprecipitated PEA-15 in U87MG

Mass spectrometry and proteomic analysis was used to identify protein interactions as previously described^60^. In this study U87MG cells were serum starved, then treated with EGF or vehicle before whole cell lysis. PEA-15-containing protein complexes were precipitated from whole cell lysates with anti-PEA15 antibodies (Pacific Immunology, Ramona, CA), or anti-HA antibodies (#51064-2-AP, Proteintech, Rosemont, IL) in the case of cells expressing HA-tagged PEA-15. Immunoprecipitants were mixed with SDS-PAGE gel loading buffer (with 5 mM DTT) and electrophoretically resolved on a 4–12% Bis–Tris NuPAGE gel (ThermoFisher). Gels were stained for 10 min with Coomassie and eight equivalently sized gel slices were excised for each sample lane and digested in-gel with sequencing grade porcine trypsin. Each digest was analyzed by nanoflow LC (Easy-nLC1000, ThermoFisher Scientific Inc.) coupled online with an Orbitrap Fusion Lumos Tribrid MS (Thermo Fisher Scientific). Following system equilibration, each sample was loaded on a C18 nano trap column, (Acclaim PepMap100 C18, 2 cm, nanoViper, Thermo Scientific) and resolved on a C18 Easy-Spray column (Acclaim PepMap RSLC C18, 2 µm, 100 Å, 75 µm × 500 mm, nanoViper, Thermo Scientific) with a linear gradient of 2% mobile phase B (95% acetonitrile with 0.1% formic acid) to 32% mobile phase B within 60 min at a constant flow rate of 250 nL/min. The C18 Easy-Spray column was heated at 50 °C during the analysis. The 12 most intense peptide ions in each MS scan were sequentially selected for high-energy collisional dissociation (HCD) using a normalized collision energy of 35%. The MS1 mass spectra were acquired at the mass range of m/z 400–1600. The Easy-Spray Ion Source (Thermo Scientific) capillary voltage and temperature were set at 2.0 kV and 275 °C, respectively. Dynamic exclusion (15 s) was enabled during the MS2 data acquisition to minimize redundant peptide fragmentation events. The RF lens was set to 30% during the MS analysis and both MS1 and MS2 spectra were collected in profile mode. Data was searched against a Swiss-Prot human protein database (http://www.uniprot.org/uniprot/) using Proteome Discoverer (v. 2.2, Thermo Fisher Scientific) via Mascot (Matrix Science Inc.) with the automatic decoy search option set followed by false-discovery rate (FDR) processing by Percolator. Data was searched with a precursor mass tolerance of 10 ppm and a fragment ion tolerance of 0.05 Da, a maximum of two tryptic miscleavages and dynamic modifications for oxidation (15.9949 mu) on methionine residues and for phosphorylation (79.9663 mu) on serine, threonine and tyrosine residues. Resulting peptide spectral matches (PSMs) were filtered using an FDR of ≤ 1% (Percolator q-value ≤ 0.01).

### Microscopy

Cells were seeded overnight on #1½ coverslips (Electron Microscopy Sciences, Hatfield, PA) in culture media supplemented with 10% serum. Cells were then fixed with 4% paraformaldehyde for 10 min followed by permeabilization with 0.1% Triton X-100. Alternatively, cells were fixed with a 1:1 mixture of methanol and acetone for 2 min. For cells adhered to fibronectin, glass coverslips were first coated with 10 µg/mL fibronectin at 37 °C for 30 min. In all cases, specimens were blocked with 1% normal goat serum (Vector Labs, Burlingame, CA). Antibodies against proteins of interest were used to stain samples, followed by incubation with goat secondary antibodies conjugated to Alexa Fluor dyes (Life Technologies, Carlsbad, CA). The following fluorochromes were used: Alexa Fluor 488 goat anti-mouse/rabbit (Green), Alexa Fluor 546 goat anti-mouse/rabbit (Red), Alexa Fluor 647 goat anti-mouse/rabbit (Blue). All coverslips were mounted with DAPI Fluoromount-G (Southern Biotech, Birmingham, AL). Phase contrast images (scratch migration and spheroid invasion assays) and epifluorescence images were acquired with a Zeiss Axiovert 200 M microscope (Zeiss, Oberkochen, Germany) and AxioVision software; 5x (Zeiss A-Plan, NA = 0.12), 10x (Zeiss A-Plan, NA = 0.25), and 100x (Olympus UPlanFLN, NA = 1.3, Olympus, Center Valley, PA) objectives were used. Confocal images were acquired on a Leica TCS SP5 confocal microscope (Leica, Wetzlar, Germany) with LAS AF software using sequential acquisition with a 63 × objective (Leica HC PL APO CS2, NA = 1.4). Total Internal Reflection Fluorescence Microscopy (TIRF) was performed on a Leica Thunder Live Cell 3D microscope with TIRF module (Leica, Wetzlar, Germany) with LASX software using a 100x/1.47 objective (part #11506318). All imaging was conducted in a room with ambient temperature maintained at 24 °C. Raw images were exported for downstream processing with ImageJ.

### In situ proximity ligation assay

Protein proximity was determined in vivo in cells using the Duolink® Proximity Ligation Assay per manufacturer’s instructions (MilliporeSigma, Bellirica, MA). U251MG cells were serum starved for 18 h then treated with 100 ng/ml EGF for 10 min. After activation with EGF cells were fixed for 15 min in 3.7% formaldehyde then washed twice with PBS. Fixed cells were permeabilized with 0.05% Triton X-100 for 10 min at RT. Fixed and permeabilized cells were then incubated in Duolink In Situ blocking solution at 37 °C for 1 h then stained with antibody to PEA-15 and either clathrin or AP-2. Detection of proteins in proximity was done according to manufacturer’s instructions and visualized using a Zeiss Axiovert 200 M microscope as described above. Quantification of the number of red dots representing interactions was done using ImageJ software.

### Immunoblotting

Lysates were resolved by SDS-PAGE. Proteins were then transferred to nitrocellulose. Primary antibodies were used to label proteins of interest. Detection was done with HRP-conjugated secondary antibodies, ECL, and film or with IRDye secondary antibodies (Li-Cor Biosciences, Lincoln, NE) and an Odyssey Infrared Imaging System (Li-Cor Biosciences, Lincoln, NE). Film scans and Odyssey scans were exported to TIF format and processed with Adobe Photoshop CS5 and Adobe Illustrator CS6.

### Isolation of endosomal fractions and phosphoproteomics in Neuroblastoma cell lines

A sub-line of adherent SMS-KCN cells, named SMS-KCN-A, was selected by culturing SMS-KCN cells on collagen coated plates and removing floating cell spheres. SMS-KCN-A cells required trypsin for passage and retained their adherent phenotype after passaging^28^. Organelle fractionation and phosphoproteomics was performed as described^28^. For organelle fractionation, cell lines were treated with ligands (LAN-6 and TrkA-CFP-expressing SK-N-BE(2): NGF, SMS-KCN:BDNF) for 10 min at 37 °C. Organelles were isolated from mechanically permeabilized cells using velocity sedimentation and flotation equilibrium centrifugation^29^, pooling two endosome fractions (E1, E2) and lysosome (Lys) and endosome fractions. Detergent-resistant (DRM) and -soluble (P1M) fractions were prepared as described^61^ except that flotation of detergent-resistant membranes was not performed for mass spectrometry experiments. Primary phosphoproteomics data for AKT substrate immunoprecipitations are available from PhosphoSitePlus (http://www.phosphosite.org) using curation set (CS) numbers 5322, 5323, 5324, 6151, 6152, 6153, 6154, 6155, 9201, 9202, 9203, 9204, 9206, 9208, 9209, 9210, 9937, 9938, 9939, 9940, 9941, 9942, 9943, 9944, 9945, and 9946. For networks, protein–protein interaction (PPI) edges from String (string.embl.de/)^62^, GeneMANIA (genemania.org/)^63^, and the kinase-substrate interactions from PhosphoSitePlus (phosphosite.org)^64^ were merged as described^65^.

Quantitative phosphoproteomic data were acquired from fractionated neuroblastoma cell lines that were subjected to treatments that activate or inhibit signaling pathways^28^. Phosphopeptides were summed for each protein (based on gene ID) or phosphorylation site (based on sequence homology) using R^66^. For statistical analysis, the absence of data was encoded as NA (data not available) instead of zero, and all samples were treated as different states in the system. Statistical relationships were embedded into three dimensional coordinates determined from dissimilarity representations based on Spearman correlation and/or Euclidean distance using t-distributed stochastic neighbor embedding (t-SNE) and clusters were defined based on proximity in these coordinates^65,67,68^. Clusters were evaluated by examination of the primary neuroblastoma phosphoproteomic data with the aid of heat maps and quantitative measures as described^65^. Clusters were then filtered and graphed as networks with protein interaction or correlation edges using RCytoscape^65,69^.

### Co-localization analysis

PEA-15 KD and scramble control cells were plated on glass coverslips and co-stained for PEA-15 and Rab5 or Rab11. Multiple ROIs of equivalent dimensions were acquired with a Leica confocal microscope, and Pearson coefficients were calculated ImageJ with the Just Another Co-localization Plugin (JACoP) following the program writer’s recommendations^70^.

### Silencing of target proteins

Stable knock down of PEA-15 in U87MG cells was done using lentivirally packaged shRNA pools of 3 targeting sequences against PEA-15 (sc-37485-V) or scrambled sequence control (sc-108080) using the manufacturer’s recommendations (Santa Cruz Biotechnology, Dallas, TX). Transduced cells were selected with 4 μg/ml puromycin dihydrochloride. Transient knock down of PEA-15 expression in U87MG was done using two independent ON-TARGETplus siRNAs with Dharmafect1 transfection reagent (Dharmacon, Lafayette, CO).

### Tumor spheroid invasion assay

Tumor spheroids were produced using U87MG scramble control and PEA-15 KD cells as previously described^71^ and embedded in a 1:1 matrigel:collagen mixture. Phase contrast images were acquired at 0, 24, and 48 h using a 5 × objective. To quantify the degree of cell invasion, phase contrast images were processed into binary images with ImageJ. Briefly, raw images were processed with the Find Edges function. An automatic threshold was then applied before measuring the area of particles from 2000 square pixels to infinity. The sum of the areas was then used to represent overall invasion.

### Scratch migration assay

As previously described, a scratch migration assay was used to measure cell motility^6^. Briefly, PEA-15 KD U87MG cells were transfected with GFP constructs. The cells were allowed to grow to confluence then scratched with a 200 µL pipette tip to form a gap. The cells were then washed in serum free media and replaced with media containing 1% serum. Images of the gap were acquired immediately after scratching (t = 0) and again after 24 h. The average closure of the gap by GFP-expressing migrating cells was measured using ImageJ software, and expressed as relative migration normalized to the t0 distance. To test for confounding effects on proliferation, baseline growth rates were measured in each transfection condition by XTT assay.

### Electric cell-substrate impedance sensing (ECIS)

The ECIS Z-Theta system and 8W1E array plates (Applied BioPhysics, Troy, NY) were used to record the electrical resistance (Ohms) due to cells migrating over an electrode. Migration rates of U87MG cells in varying experimental conditions were estimated from these resistance changes over time. U87MG cells were transfected 24 h before seeding 60,000 cells into fibronectin-coated wells of the 8W1E plates. When a monolayer of cells was established approximately 24 h after seeding, cells were wounded by applying 15 s of 2 mA electrical current at 40 kHz. As a result, the area directly over the electrode was free of cells, and resistance recordings dropped significantly. As cells of the surrounding monolayer begin to migrate onto the empty electrode, impedance levels increase accordingly. An unwounded sample was included to track each of the experimental groups’ progress toward recovery, which was reached when the resistance levels aligned with that of the corresponding unwounded sample (control). At the same time point, resistance levels of each experimental group were divided by resistance levels of the control, and a ratio value of 1 was indicative of full recovery. To estimate cell migration rate, we used 60% of the electrode radius as the threshold distance (0.6*125 µm = 75 µm). The then calculated migration rate by dividing 75 µm by the time it took the cells to cover that distance. This yield a measurement of cell migration speed with units of µm/hr.

ECIS chambers were also used to evaluate U87MG cell adhesion. Baseline resistance levels of both scramble and PEA-15 shRNA U87MG cells were taken after the establishment of a cell monolayer on the 8W1E plates and immediately before wounding. Differences in resistance were interpreted as changes in adhesion and cell spreading on the electrode surface.

### Adhesion assay

Fibronectin-coated 96 well culture plates were seeded with 50,000 cells in serum-free media. Cells were allowed to adhere for 30 min then washed twice with phosphate buffered saline. Remaining attached cells were then stained with 0.1% Crystal Violet in 2% ethanol. Cells were then washed with distilled water and air-dried. Crystal Violet is then extracted with 10% acetic acid and quantified by measuring absorbance at 590 nm.

### Integrin tail pulldown assay

Recombinant His-tagged integrin tails (Mark Ginsberg, UCSD) were produced and purified as previously described^72,73^ then immobilized on His-bind resin (Millipore, Billerica, MA ). HeLa cells were transfected with the indicated constructs then lysed in XTT lysis buffer. Cell lysates were then incubated with integrin tails for 24 h, followed by boiling in SDS-PAGE loading buffer. Precipitated PEA-15 was detected by Western blot, and integrin tail expression was detected by coomassie stain.

### Integrin internalization assay

Integrin trafficking was quantified using an established biotinylation approach^14^. U87MG glioma cells are seeded for approximately 50% confluency. Cells are then serum starved for 30 min in 1% serum, followed by wash in cold DPBS and surface biotinylation with 0.2 mg/mL Sulfo-NHS-SS-Biotin (Thermo Fisher) for 20 min at 4 °C. Cells are washed once then incubated with pre-warmed 1% serum at 37 °C in the presence of 75 µM Primaquine diphosphate (MP Biomedicals, Santa Ana, CA). At each time point cells are returned to ice and washed with cold DPBS to stop internalization. Biotin remaining on the surface is removed by incubation with 20 mM MESNA (sodium 2-mercaptoethanesulfonate) (Sigma-Aldrich, St. Louis, MO) reducing agent for 15 min at 4 °C. MESNA is then quenched with 20 mM Iodoacetamide (Sigma-Aldrich) for 10 min at 4 °C, followed by washes and cell lysis with lysis buffer (25 mM Tris–HCl, pH 7.4, 100 mM NaCl, 2 mM MgCl_2_, 0.5 mM EGTA, 1% Triton X-100, 5% glycerol, protease inhibitor cocktail, 1 mM Na_3_VO_4_ and 1 mM phenylmethanesulfonylfluoride (PMSF)). Lysates were corrected to equal protein concentration and applied to capture ELISA or to western blotting.

### Capture ELISA

Nunc Maxisorp 96-well plates were coated with 50 µL of mouse anti-integrin α5 monoclonal antibodies (#555651, BD Biosciences, San Jose, CA) diluted to 5 µg/mL in carbonate/bicarbonate coating buffer (pH 9.2) overnight at 4 °C. The plate was washed with PBST (PBS with 0.05% Tween-20) and blocked with 5% BSA for 1 h at room temperature. 50 µL of lysate is loaded in triplicate and incubated overnight at 4 °C. The plates are then washed and incubated with 50 µL HRP-conjugated streptavidin (Thermo Fisher) for 1 h at 4 °C followed by generous washing and color development with OPD (*o*-phenylenediamine dihydrochloride) (Thermo Fisher). The reaction was stopped with 2.5 M sulfuric acid. Absorbance was measured with a plate reader at 490 nm. Integrin internalization is then calculated as a ratio of MESNA-resistant signal to starting surface signal (%Internalization).

### Integrin recycling assay

To measure integrin recycling, cells are prepared as above for internalization assays. After surface biotinylation, cells are incubated at 37 °C for 30 min followed by surface stripping with MESNA. Internalized integrins are then chased back to the membrane with prewarmed 10% serum. This was followed by a second round of MESNA to remove recycled biotin. Cells are then quenched and lysed then applied to capture ELISA. Recycling is calculated as the percent-reduction of post-chase signal from pulse signal.

### Statistical analysis

Each experiment was performed independently three or more times as indicated. Statistics were performed on biological replicates. The statistical significance of the differences in biological replicates was measured by unpaired *Students’* t-test and *p* values are reported.

## Supplementary Information

Supplementary Table 1.

Supplementary Table 2.

Supplementary Figures.

## Data Availability

All original data supporting the reported findings are available from the corresponding author upon reasonable request.
