# Supplementary material for: Proteomics analysis identifies PEA-15 as an endosomal phosphoprotein that regulates α5β1 integrin endocytosis
Source: Sci Rep. 2021 Oct 6;11:19830. doi: 10.1038/s41598-021-99348-z (PMC8494857; doi:10.1038/s41598-021-99348-z)
Supplement: Supplementary file 3 — Supplementary Figures. [file 41598_2021_99348_MOESM3_ESM.pdf]

Proteomics analysis identifies PEA-15 as an endosomal phosphoprotein that regulates α5β1 integrin endocytosis

Maisel J. Caliva (1), Won Seok Yang (1), Shirley Young-Robbins (1), Ming Zhou (2), Hana Yoon (1), Michelle L. Matter (1), Mark L. Grimes (3), Thomas Conrads (2), Joe W. Ramos* (1)

(1) Cancer Biology Program University of Hawaii Cancer Center
University of Hawaii at Mānoa
701 Ilalo Street
Honolulu, HI 96813, USA

(2) Women's Health Integrated Research Center at Inova Inova Women's Service Line
Inova Health System
3289 Woodburn Rd, Suite 375,
Falls Church, VA, 22003, USA

(3) Division of Biological Sciences
Center for Structural and Functional Neuroscience University of Montana
32 Campus Drive
Missoula, MT 59812, USA

* Corresponding Author:
Joe William Ramos, PhD
Cancer Biology Program
University of Hawaii Cancer Center
University of Hawaii at Manoa
701 Ilalo Street, Honolulu, HI 96813
USA Voice: (808) 564-5843;
Fax: (808) 587-0742
E-mail: joeramos@hawaii.edu

Supplemental. Phospho-PEA-15 is enriched in neuroblastoma endosomal fractions

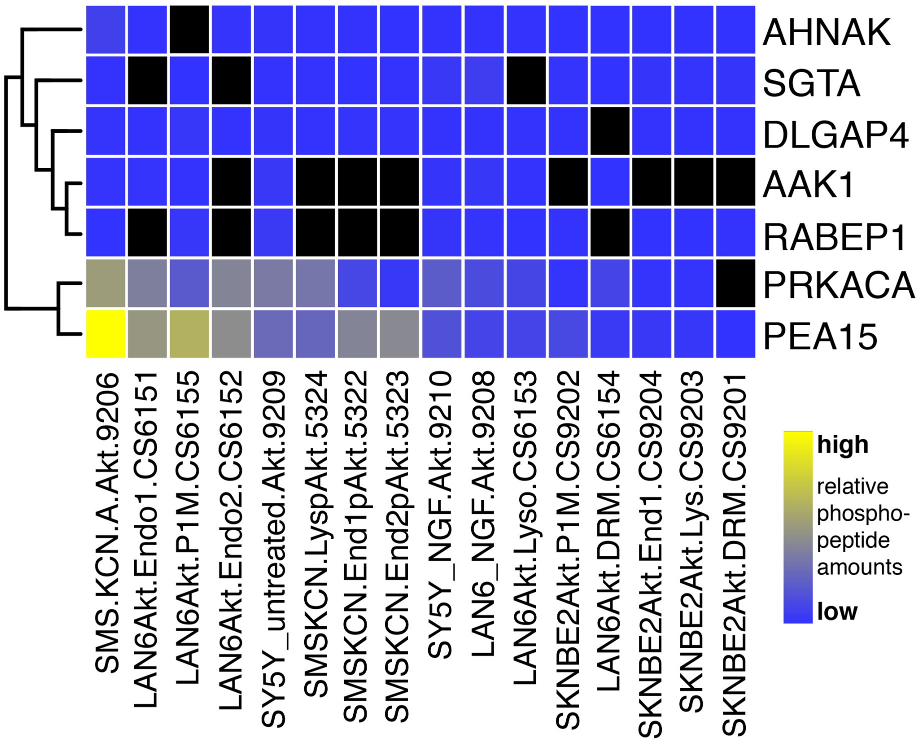

Supplemental Figure 1. Phospho-PEA-15 is enriched in neuroblastoma endosomal fractions. Heat map displays the relative total phosphopeptide amounts for each protein on a blue-yellow scale (black represents NA; key, bottom left), sorted most to least left to right and top to bottom for samples; rows (proteins) were sorted by hierarchical clustering using a modified distance function that handles missing values. Phosphorylated PEA-15 was most strongly detected in SMS-KCN cells selected for adherence to collagen (SMS-KCN-A) and also detected in SH-SY5Y cells and endosome (End1, End2), lysosome (Lys), detergent-soluble (P1M) and detergent-resistant (DRM) fractions isolated from three neuroblastoma cell lines, LAN-6, SMS-KCN, and SK-N-BE(2).

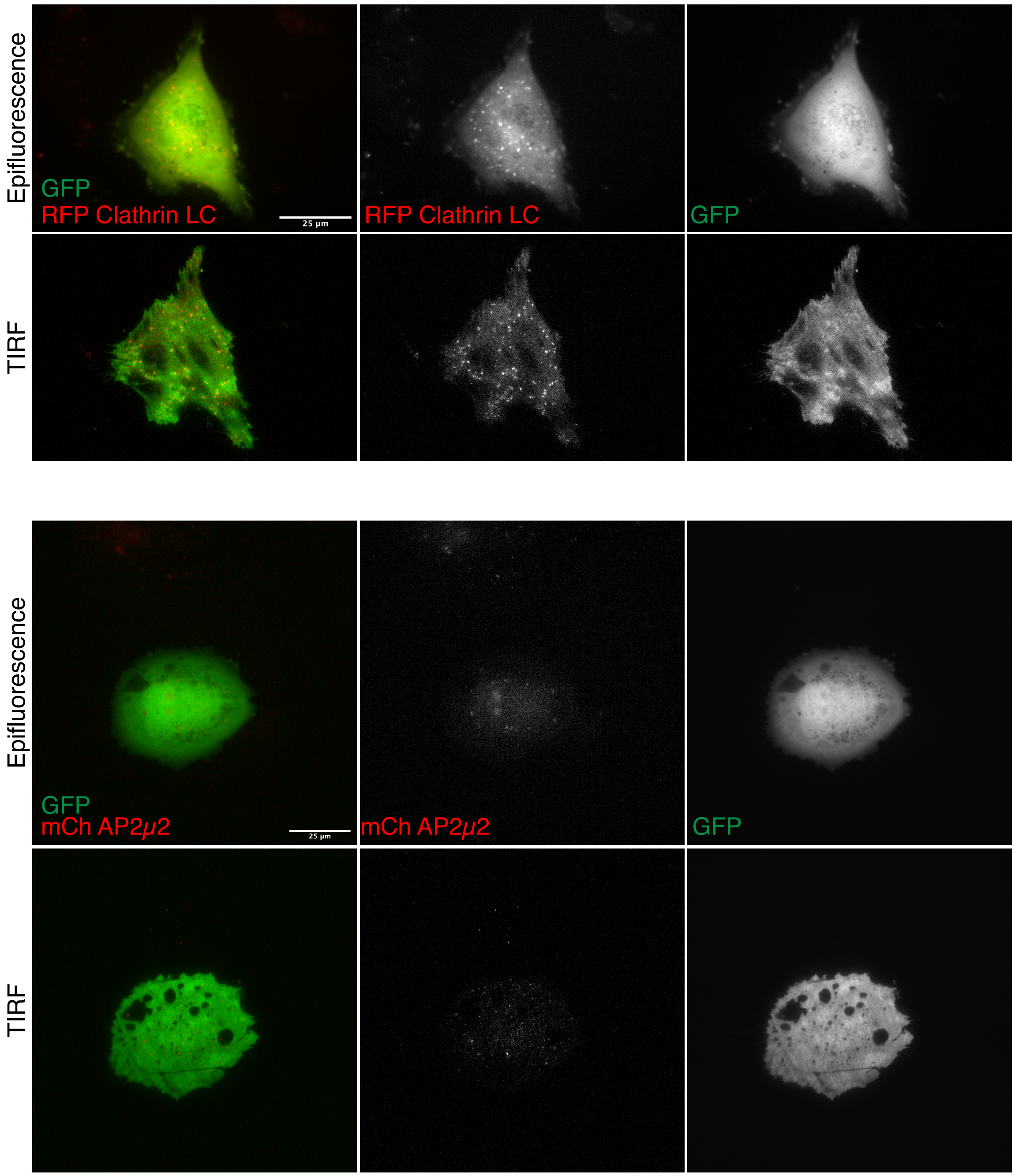

Supplemental Figure 2. GFP control for TIRF microscopy. RFP-tagged Clathrin Light Chain or mCherry-tagged AP2μ2 was co-expressed with GFP in U87MG cells. Epifluorescent and TIRF images were acquired. Scale bars = 25 μm.

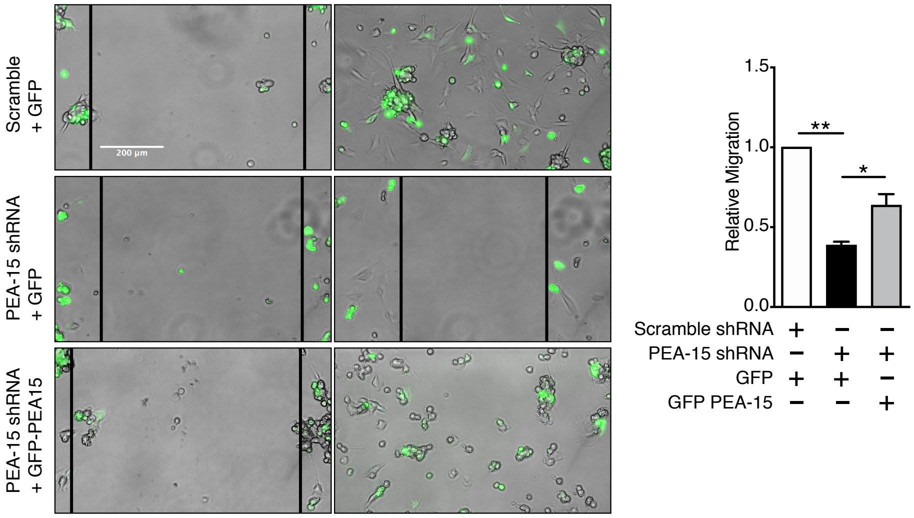

Supplemental Figure 3. PEA-15 is required for cell migration (scratch assay). U87MG PEA-15 KD cells were transfected with GFP or GFP-tagged PEA-15. Confluent layers were then scratched and relative cell migration of GFP expressing cells was monitored over time (**p<0.005, *p<0.05, n = 3, Scale bar = 200 μm).

Supplemental Table 1. < Supp Table 1 PEA15_U87_comparison_coverage.xlsx>

Supplemental Table 2. < Supp Table 2 U87HA_PEA15_comparison_coverage.xlsx>

Supplemental Western Blot / SDS-PAGE Scans

Figure 1C – Western blot: Clathrin Heavy Chain
< 5-31-2019 IP_PEA15 IB_CHC.tif>

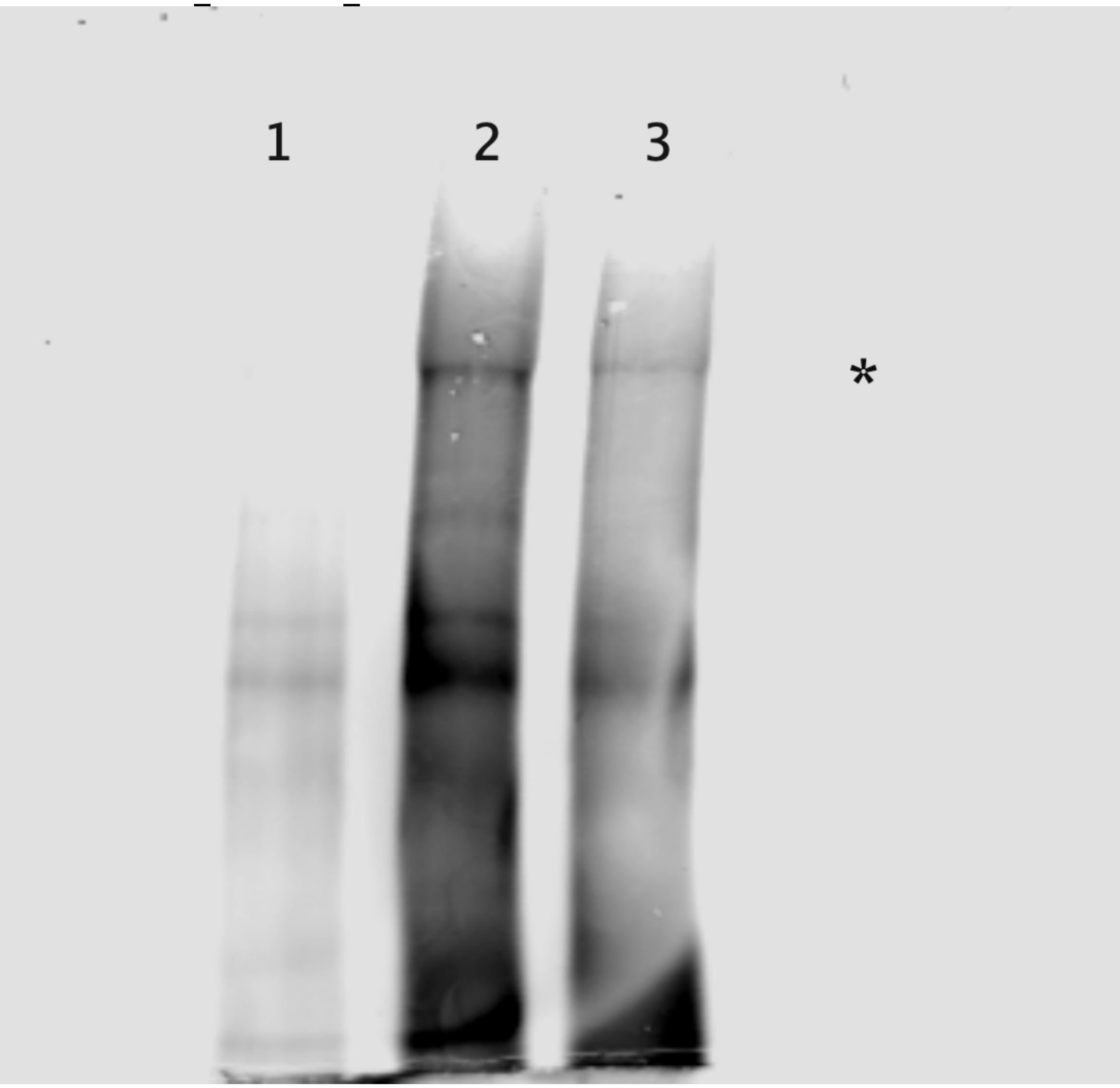

Lanes 1 and 2 are included in the final figure. *Denotes the position of the signal of interest.

Figure 1C – Western blot: PEA-15
< 5-31-2019 IP_PEA15 IB_PEA15.tif>

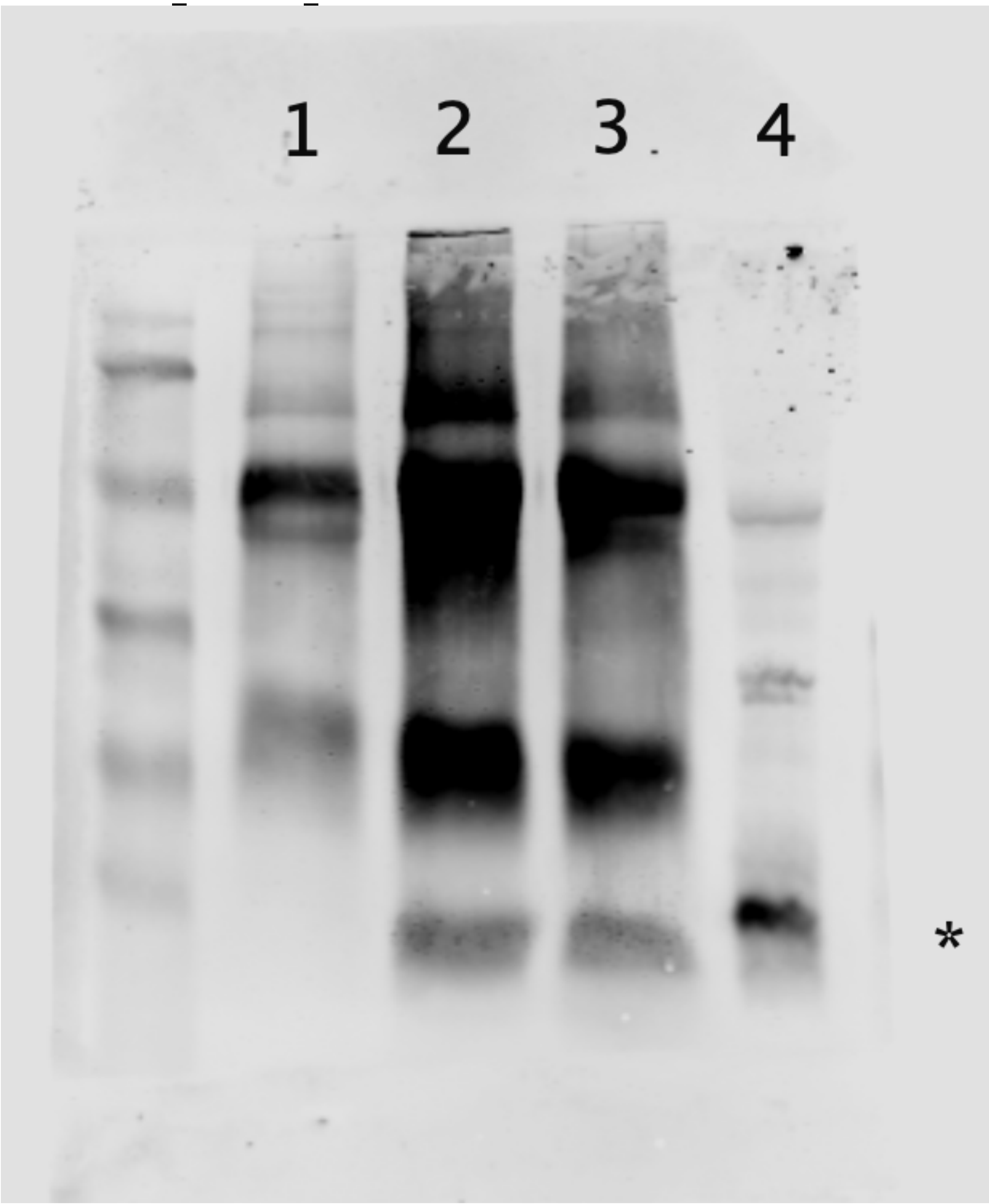

Lanes 1 and 2 are included in the final figure. Lane 4 was used for the Input lane for PEA-15. *Denotes the position of the signal of interest.

Figure 1C – Western Blot: Clathrin Heavy Chain
< 5-31-2019 IP_PEA15 IB_CHC INPUT.tif>

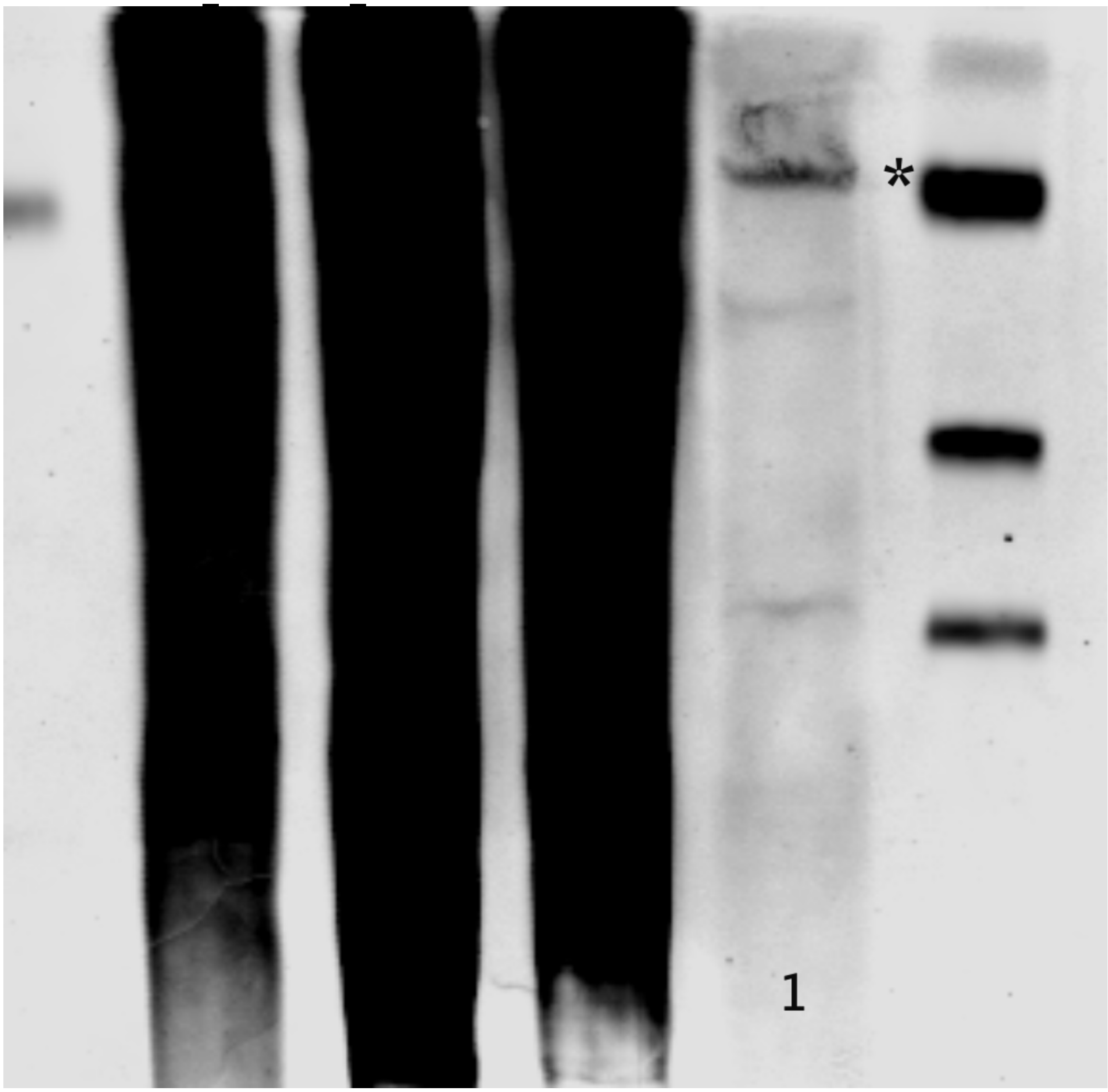

Lane 1 was used for the Input lane for Clathrin Heavy Chain. *Denotes the position of the signal of interest.

Figure 5A – Western blot: PEA-15
<Green_PEA15.tif (green).tif>

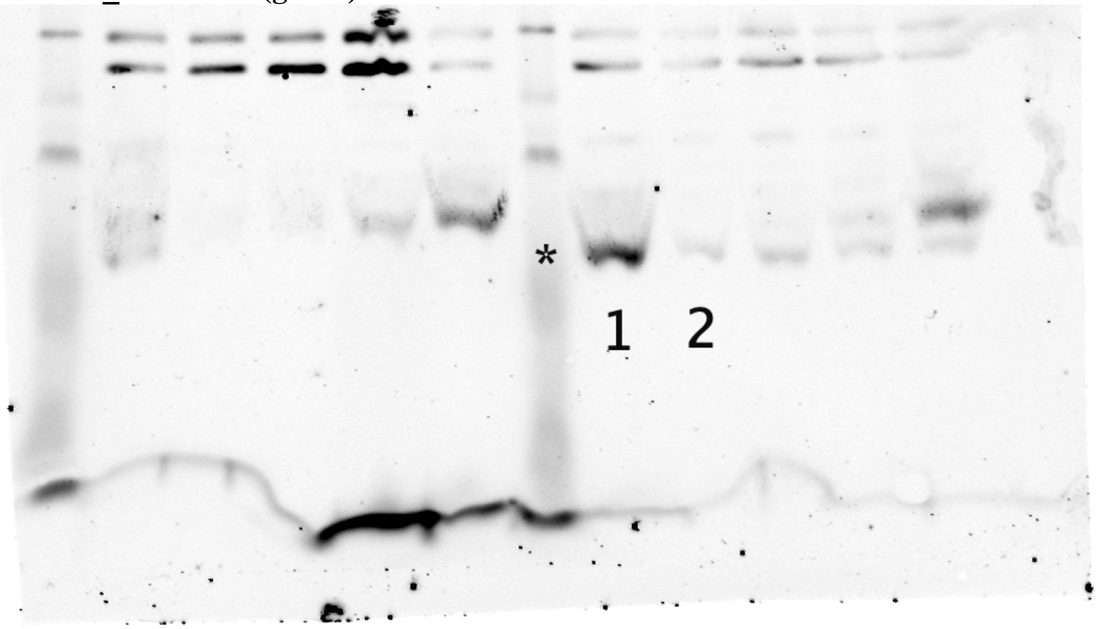

Lanes 1 and 2 are included in the final figure. *Denotes the position of the signal of interest.

Figure 5A – Western blot: Tubulin
<Green_Tubulin.tif (green).tif>

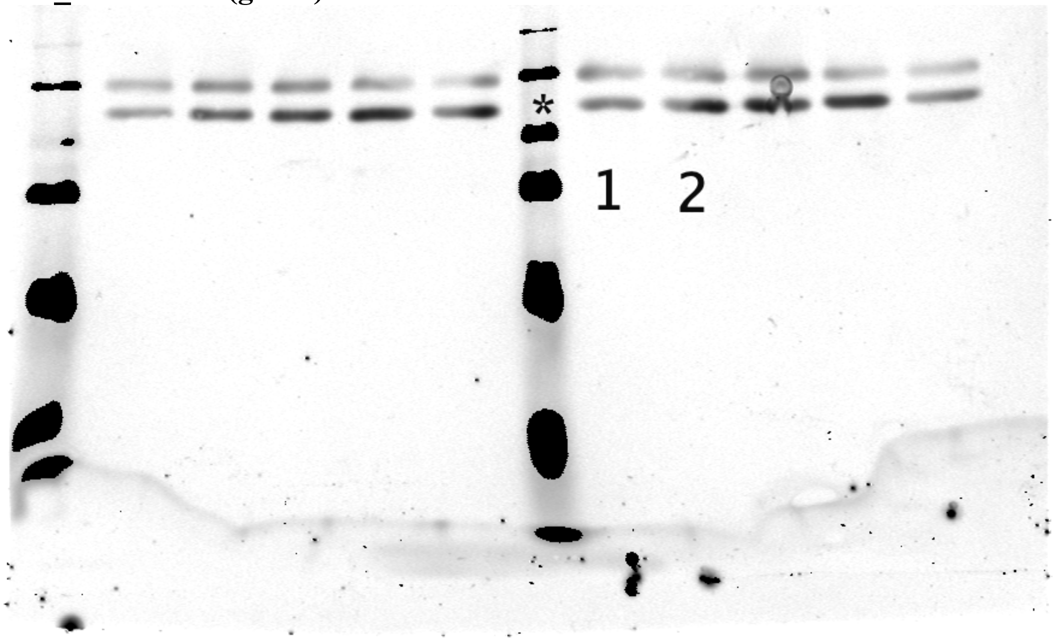

Lanes 1 and 2 are included in the final figure. *Denotes the position of the signal of interest.

Figure 6D – Western Blot: HA-tag (PEA-15)
<Inputs.tif>

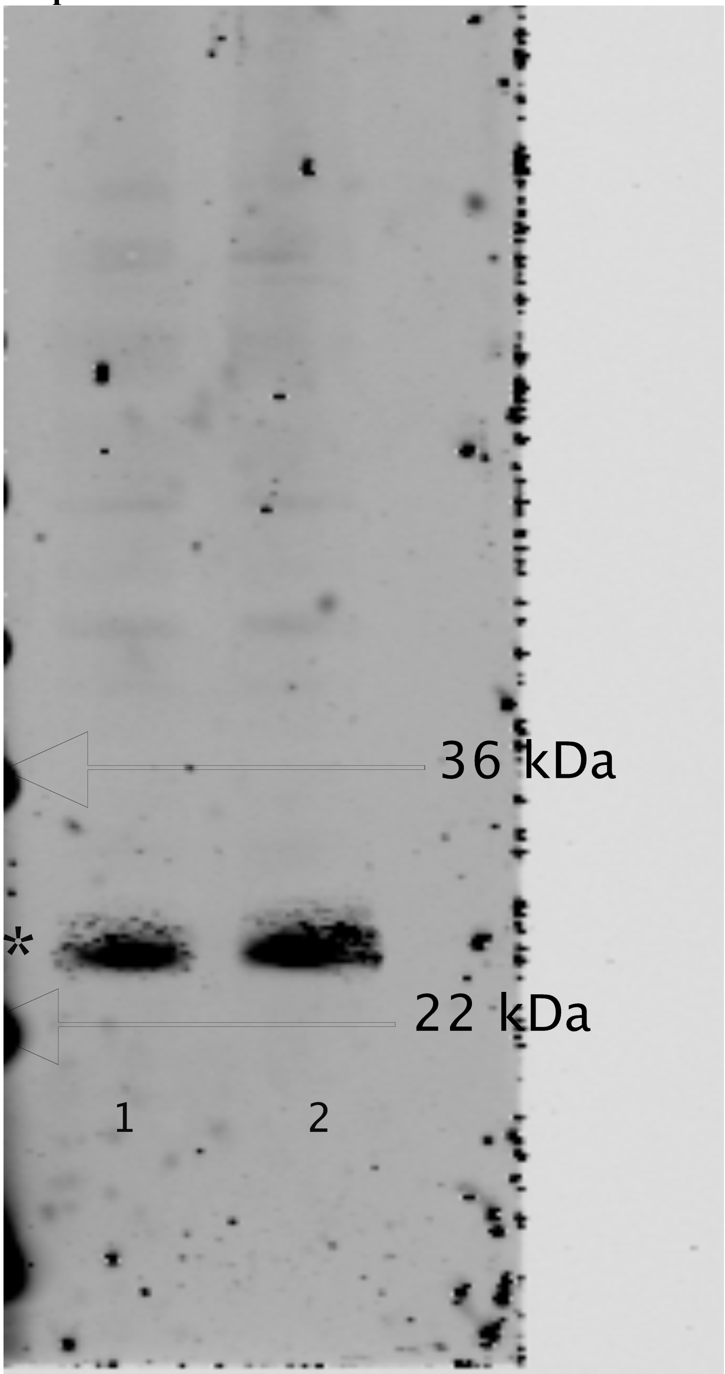

Lane 1 was used for the Input signal in the final figure. *Denotes the position of the signal of interest.

Figure 6D – Western Blot: HA-tag (PEA-15)
<Integrin PD.tif>

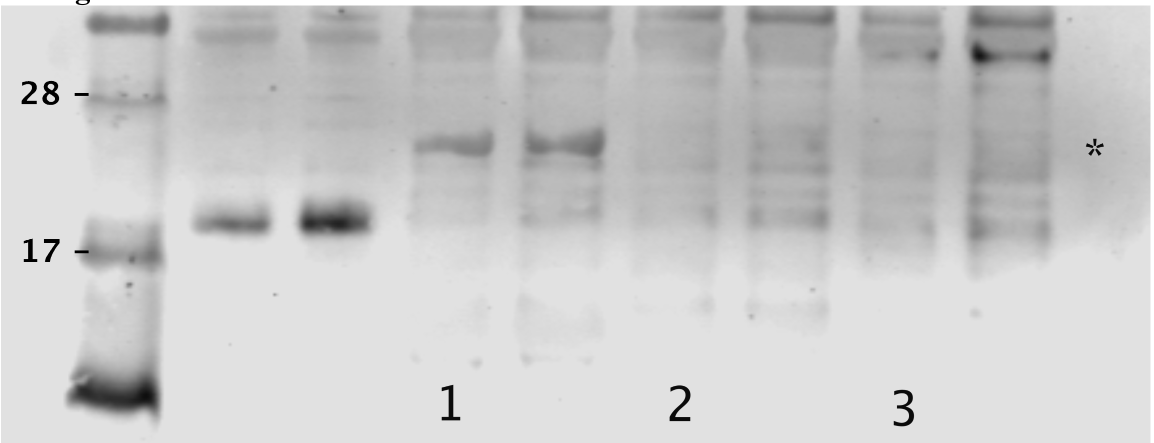

Lane 1 (β1 tail pulldown), lane 2 (NPXA pulldown), and lane 3 (αIIb pulldown) were used in the final figure. *Denotes the position of the signal of interest.

Figure 6D – Coomassie stain (confirming Integrin tail expression)
<FCM_0484_2014-06-09_16-47-26.tif>

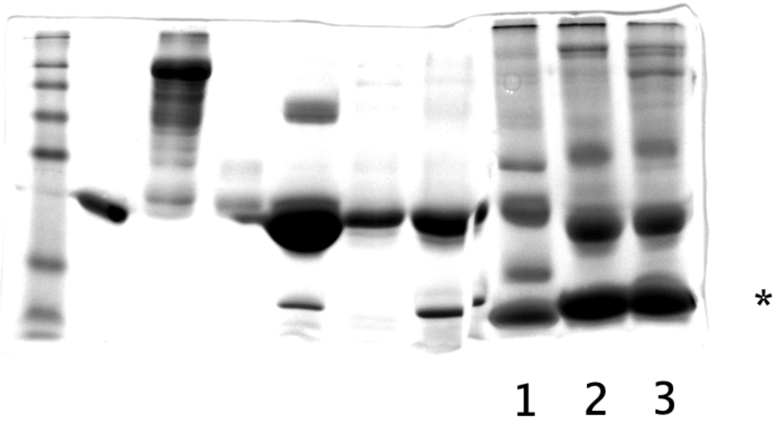

Lane 1, 2, and 3 were used for the final figure. *Denotes the position of the signal of interest.

Figure 8A – Western Blot: PEA-15
<10-4-2019 Green_PEA15 HQ.tif (green).tif>

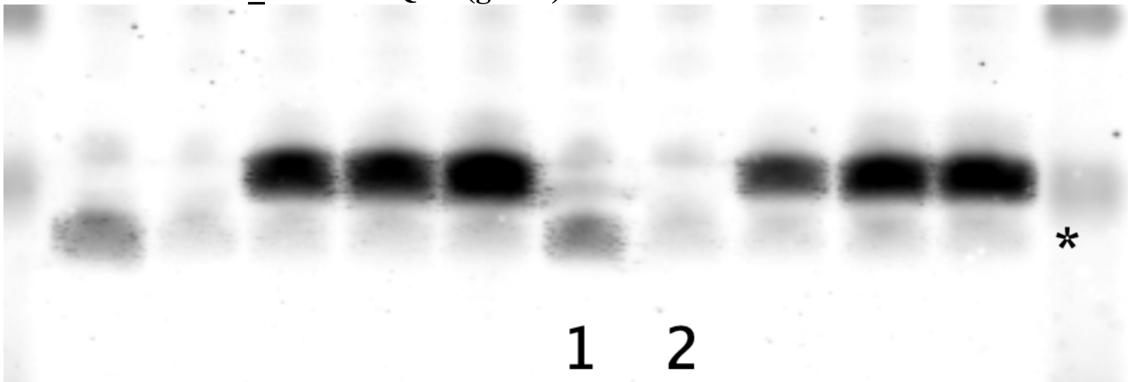

Lanes 1 and 2 are included in the final figure. *Denotes the position of the signal of interest.

Figure 8A – Western Blot: α5 Integrin
<10-7-2019 Red_Alpha5Integrin HQ.tif (red).tif>

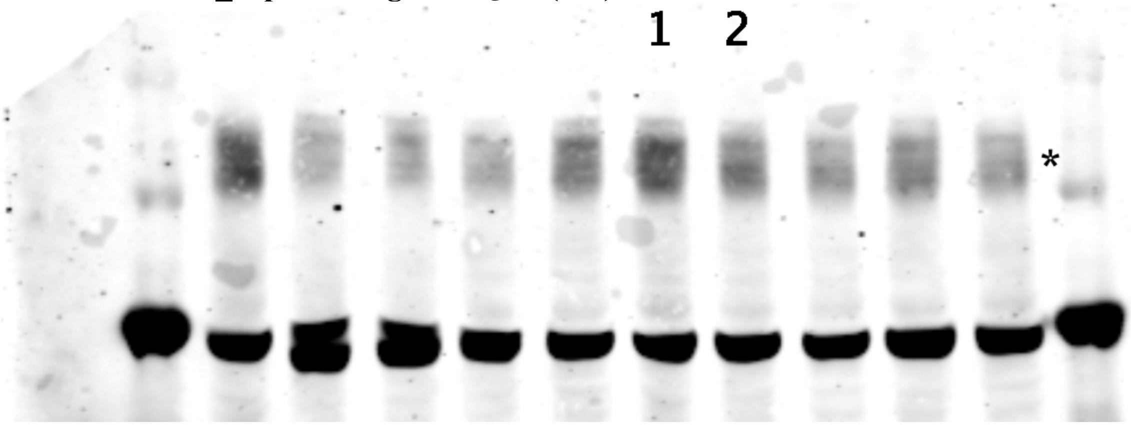

Lanes 1 and 2 are included in the final figure. *Denotes the position of the signal of interest.

Figure 8A – Western Blot: β1 Integrin
<10-7-2019 Green_Beta1Integrin HQ.tif (green).tif>

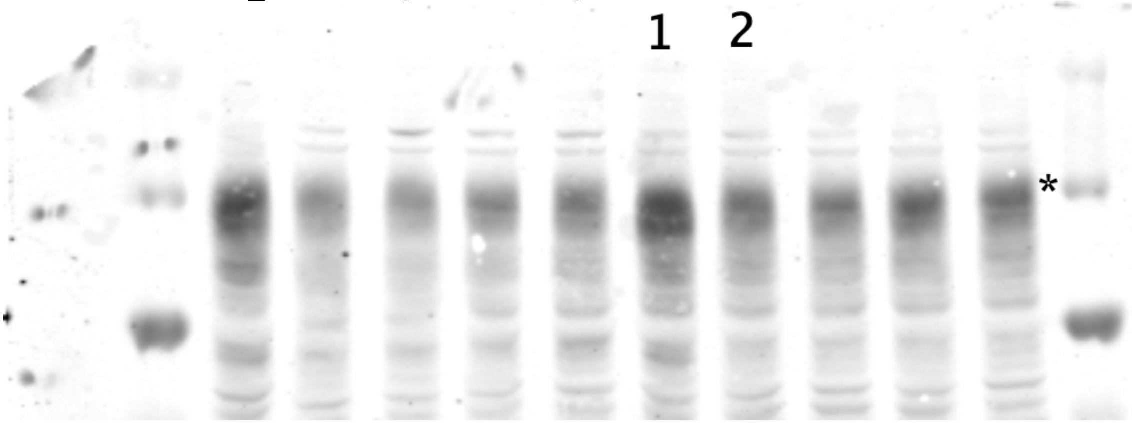

Lanes 1 and 2 are included in the final figure. *Denotes the position of the signal of interest.

Figure 8A – Western Blot: Rab5
<10-7-2019 Green_Rab5 HQ.tif (green).tif>

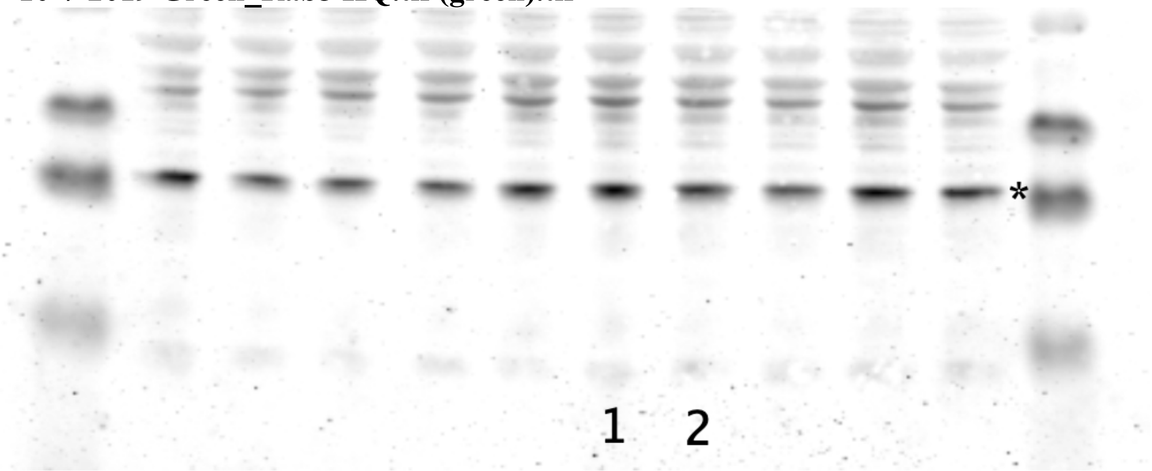

Lanes 1 and 2 are included in the final figure. *Denotes the position of the signal of interest.

Figure 8A – Western Blot: Clathrin Heavy Chain
<10-2-2019 Green_CHC HQ.tif (green).tif>

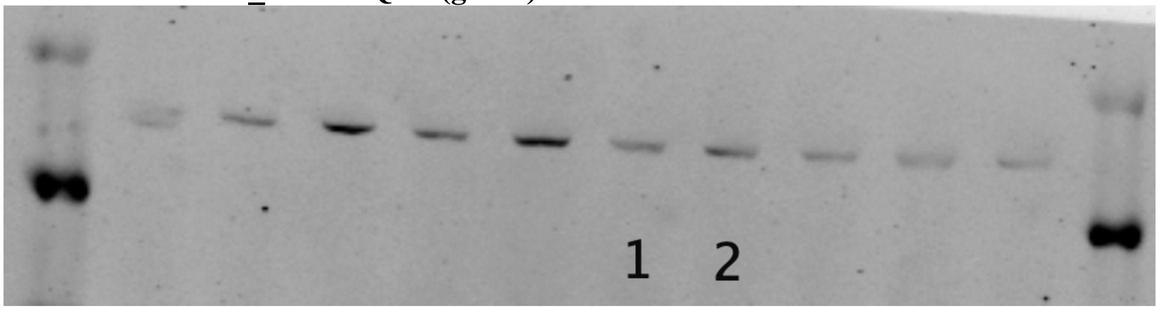

Lanes 1 and 2 are included in the final figure.

Figure 8A – Western Blot: Tubulin
<10-2-2019 Red_aTubulin HQ.tif (red).tif>

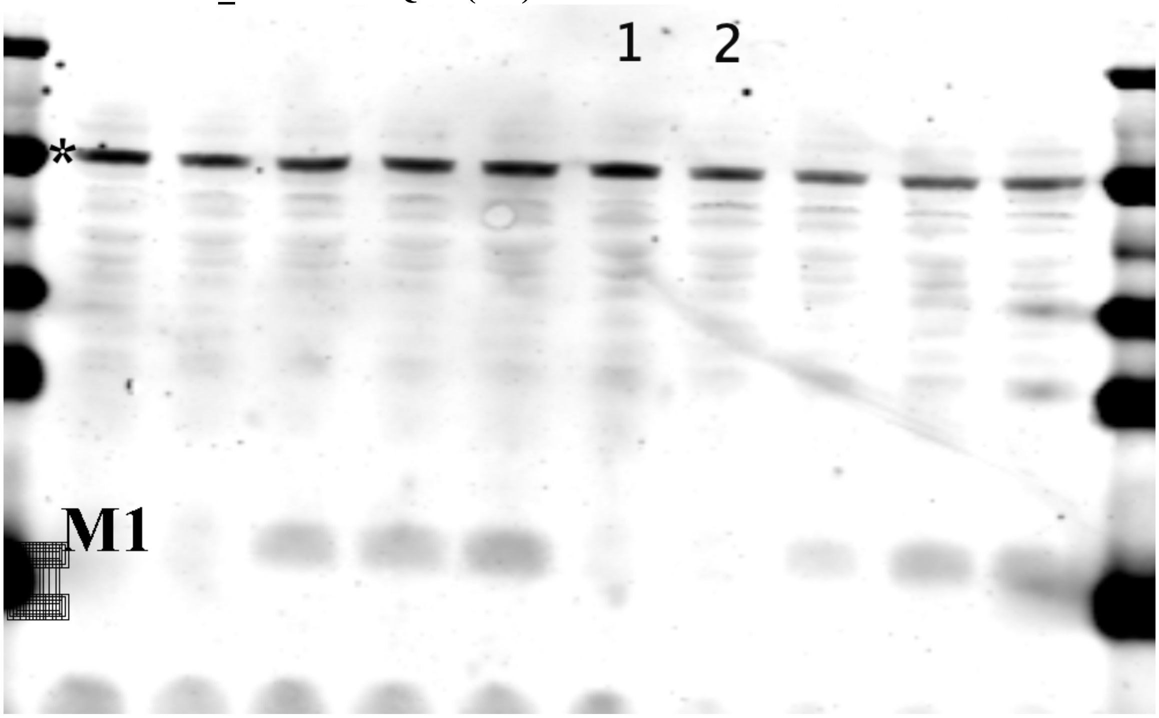

Lanes 1 and 2 are included in the final figure. *Denotes the position of the signal of interest.

Figure 8C – Western Blot: PEA-15
<3-6_7-2020 QC Blot Gr_PEA15 (green).tif>

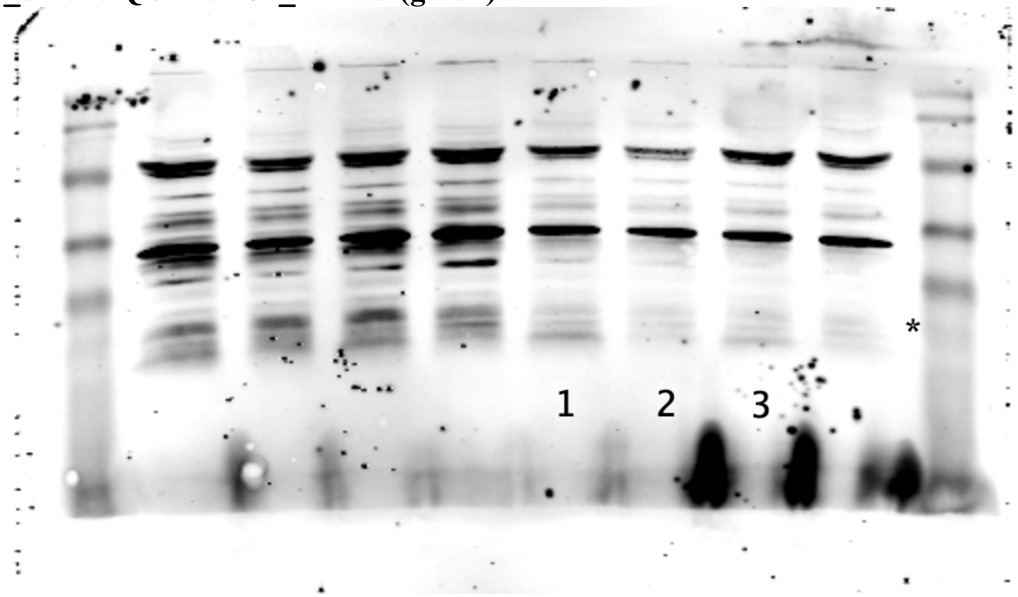

Lanes 1, 2, and 3 were included in the final figure. *Denotes the position of the signal of interest.

Figure 8C – Western Blot: β1 Integrin
<3-6_7-2020 QC Blot Gr_Beta1.tif (green).tif>

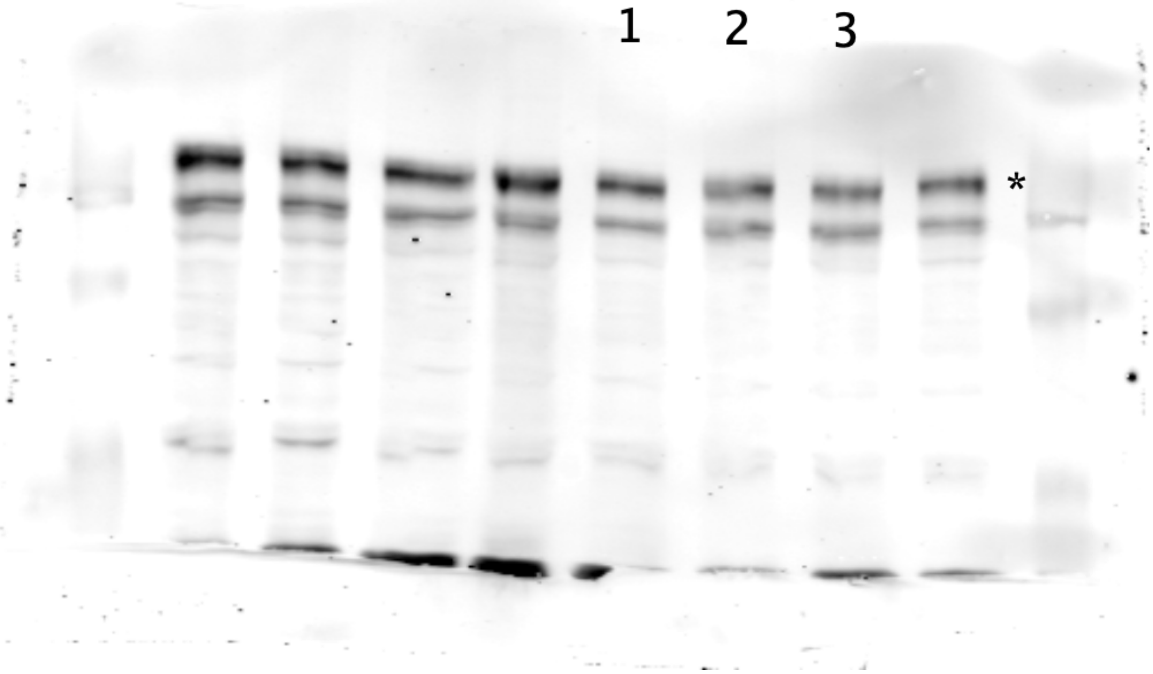

Lanes 1, 2, and 3 were included in the final figure. *Denotes the position of the signal of interest.

Figure 8D – Western Blot: Streptavidin (Biotinylated - β1 Integrin)
<biorad 2012-05-12 16hr 58min.tif>

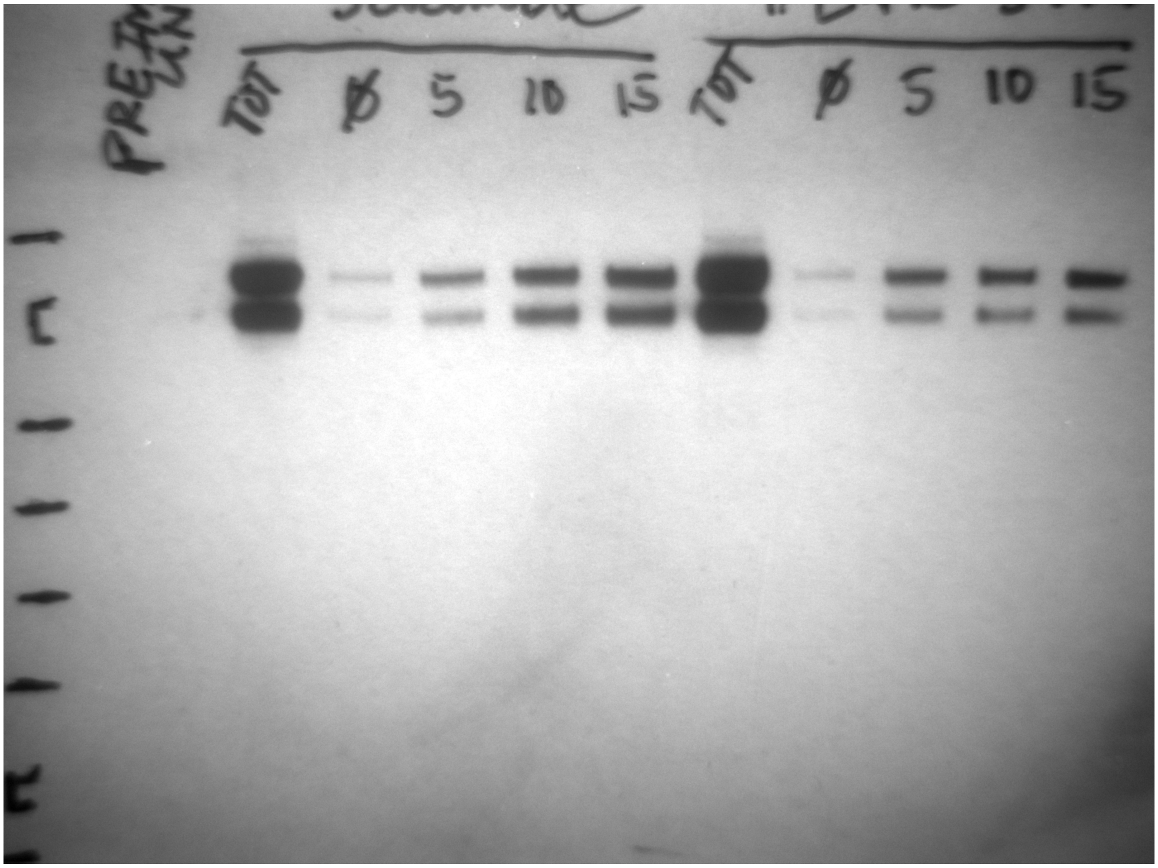

Figure 8D – Western Blot: β1 Integrin
<800 Beta1.TIF>

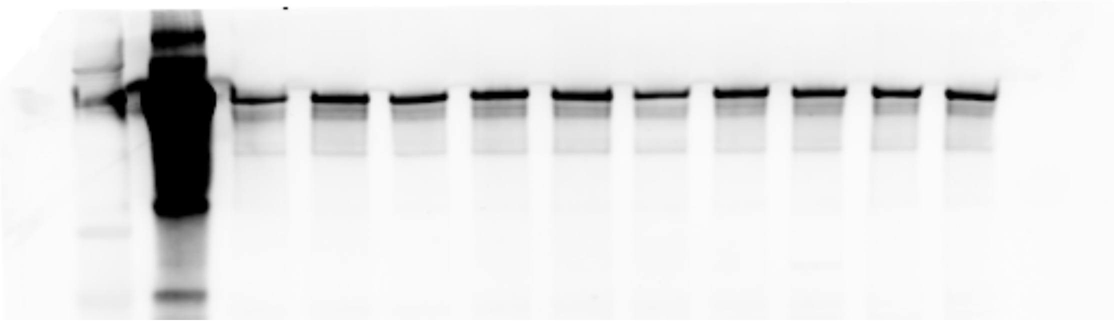

Figure 8F – Western Blot: HA-Tag (PEA-15)
<HA_PEA15.tif>

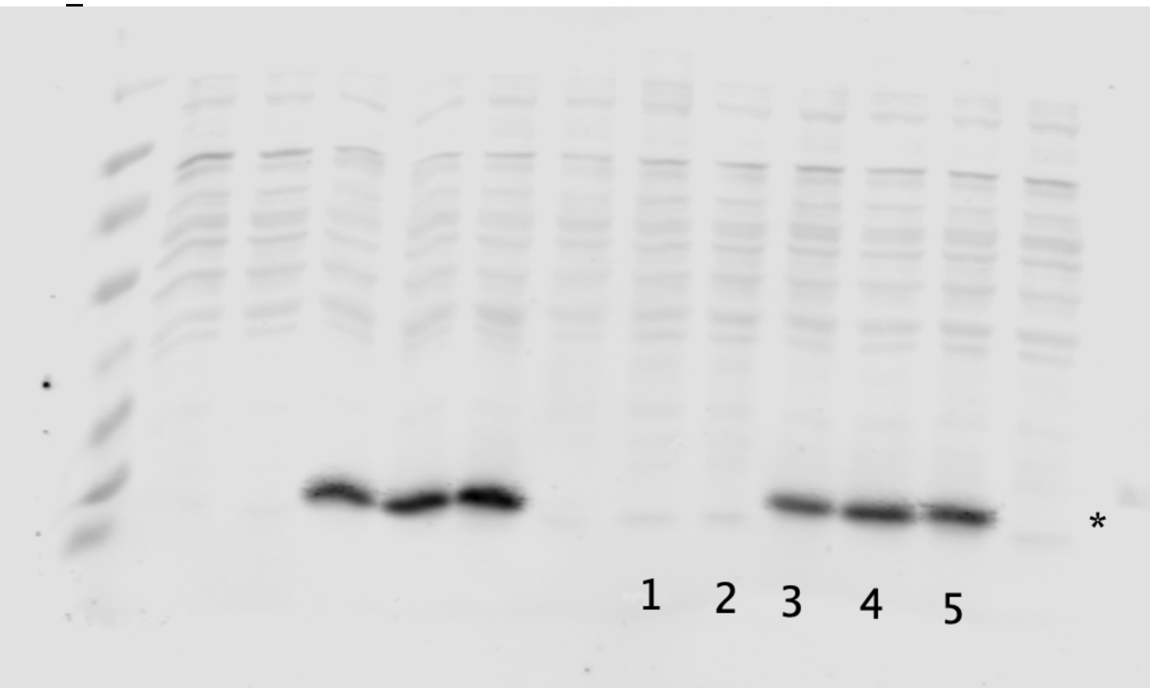

Lanes 1 through 5 were included in the final figure. *Denotes the position of the signal of interest.

Figure 8F – Western Blot: Tubulin
<Tubulin.tif>

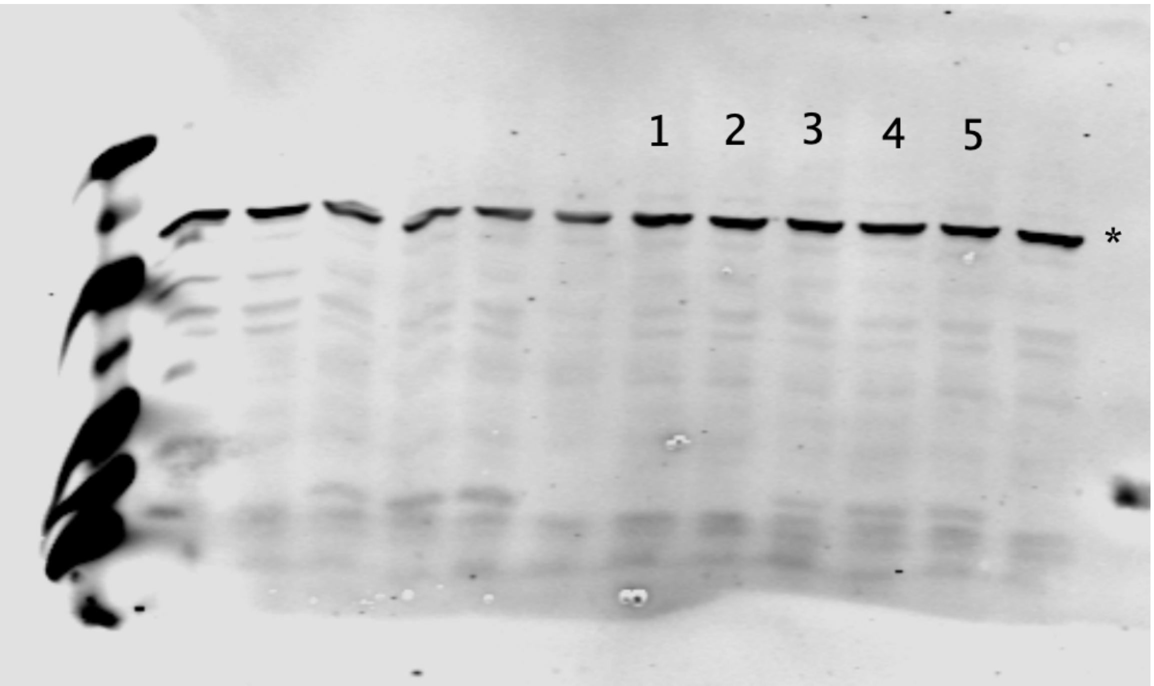

Lanes 1 through 5 were included in the final figure. *Denotes the position of the signal of interest.
